# Multi-Target Directed Ligands (MTDLs) Binding the σ_1_ Receptor as Promising Therapeutics: State of the Art and Perspectives

**DOI:** 10.3390/ijms22126359

**Published:** 2021-06-14

**Authors:** Francesca Serena Abatematteo, Mauro Niso, Marialessandra Contino, Marcello Leopoldo, Carmen Abate

**Affiliations:** Dipartimento di Farmacia-Scienze del Farmaco, Università degli Studi di Bari ALDO MORO, Via Orabona 4, 70125 Bari, Italy; francesca.abatematteo@uniba.it (F.S.A.); mauro.niso@uniba.it (M.N.); marialessandra.contino@uniba.it (M.C.); marcello.leopoldo@uniba.it (M.L.)

**Keywords:** sigma receptors, sigma-1 receptor, multi-target directed ligands (MTDLs), sigma-1 ligands, polypharmacology

## Abstract

The sigma-1 (σ_1_) receptor is a ‘pluripotent chaperone’ protein mainly expressed at the mitochondria–endoplasmic reticulum membrane interfaces where it interacts with several client proteins. This feature renders the σ_1_ receptor an ideal target for the development of multifunctional ligands, whose benefits are now recognized because several pathologies are multifactorial. Indeed, the current therapeutic regimens are based on the administration of different classes of drugs in order to counteract the diverse unbalanced physiological pathways associated with the pathology. Thus, the multi-targeted directed ligand (MTDL) approach, with one molecule that exerts poly-pharmacological actions, may be a winning strategy that overcomes the pharmacokinetic issues linked to the administration of diverse drugs. This review aims to point out the progress in the development of MTDLs directed toward σ_1_ receptors for the treatment of central nervous system (CNS) and cancer diseases, with a focus on the perspectives that are proper for this strategy. The evidence that some drugs in clinical use unintentionally bind the σ_1_ protein (as off-target) provides a proof of concept of the potential of this strategy, and it strongly supports the promise that the σ_1_ receptor holds as a target to be hit in the context of MTDLs for the therapy of multifactorial pathologies.

## 1. Introduction

The most exploited paradigm in medicinal chemistry is the “one-target, one-disease”, according to which ligands are developed to act toward a single target in order to exert a beneficial effect. This kind of approach, even if largely applied, may lead to unsuccessful results. Indeed, pathologies are mostly based on tangled mechanisms that involve different kinds of targets. Thus, a single target approach could be inappropriate because of its impossibility to produce an effect on the different pathways associated with the pathology.

On the basis of this idea, a multi-target strategy has been proposed in order to hit at the same time the diverse targets involved and modulate the diverse mechanisms that underlie the pathology.

The first step of this multi-target strategy has been the co-administration of more drugs all together, with the aim of obtaining a synergic effect. Some examples of this approach are represented by current therapeutic programs. Pathologies such as hypertension, cancer, neurodegeneration, AIDS, and depression can require the administration of up to three different classes of drugs.

Although this approach has therapeutic beneficial effects, the higher the number of drugs administered, the higher the number of metabolites that could produce side effects. Moreover, the co-administration of different drugs can produce cross-resistance to the therapeutics and inconvenient pharmacokinetic profiles. With the aim to overcome such problems, the multi-target directed ligand (MTDL) approach has been introduced. This strategy is based on the administration of a single drug with multiple effects because such a drug incorporates two or more pharmacophores in a single molecule. This incorporation can produce *hybrid drugs* or *chimeric drugs*. *Hybrid drugs* are obtained when two or more pharmacophores are connected using a stable or metabolizable linker. These structures not only have a high molecular weight, which is detrimental for oral bioavailability, but they are also very flexible, so that binding to the targets can be reduced. On the other hand, *chimeric drugs* are obtained by fusing or merging two or more different pharmacophores into one. In contrast to the former type, *chimeric drugs* have better pharmacokinetic properties and better interaction with targets because of their rigidity [1,2].

The synthesis of MTDLs can follow a *knowledge-based approach* or a *screening approach*. The former approach is based on the use of diverse and focused libraries of molecules and relies on well-known drugs and compounds. In the *screening approach* instead, ligands that demonstrate a solid affinity for a certain target are explored in their activities/affinities for other targets. However, in both cases, a fine work of optimization is needed to obtain the best compromise that guarantees the interaction with all targets [3].

In this review, the authors want to discuss the use of the MTDL approach that exploits the interaction with sigma (σ) receptors. In fact, even if these receptors are still not completely understood, their involvement in several pathologies has been widely demonstrated, so that the synergy obtained by a simultaneous interaction with σ receptors and other biological targets appears as a beneficial strategy to face multifactorial pathologies.

σ Receptors were discovered in 1976, when studies performed on opioid receptors revealed the presence of proteins that do not bind **naloxone** and **etorphine** but display high affinity for (**+**)-**pentazocine**, which is still used in binding studies [4]. A milestone in the history of σ receptors has been attained when two different protein subtypes were discovered, namely σ_1_ and σ_2_ receptors [5].

## 2. σ1 Receptors

σ_1_ Receptors have been found in the central nervous system (CNS), particularly in the granular layer of the olfactory bulb, in many cortical layers, and in the dentate gyrus [6], where they exert their most important activities. A slightly lower expression has been also found in some pyramidal layers of the hippocampus, various hypothalamic nuclei, the septum, the central gray, the motor nuclei of the hindbrain, and the dorsal horn of the spinal cord [6]. At the cellular level, σ_1_ receptors have been found in ependymocytes, which border the ventricular compartments, and in neurons located within the CNS parenchyma [6].

In peripheral organs, σ_1_ receptors have been found in the gastrointestinal tract [7], vas deferens [8], in liver and kidney [9], heart [10], adrenal medulla, pituitary, testis, and ovaries [11].

The σ_1_ receptor is localized at the mitochondrial-associated endoplasmic reticulum (ER) membranes (MAM) in association with the binding immunoglobulin protein (BiP) in a resting state. Upon agonist stimulation, or Ca^2+^ depletion, the σ_1_ receptor dissociates from BiP and stabilizes inositol 1,4,5-trisphosphate receptor type 3 (IP3R3) at MAM, increasing Ca^2+^ transfer from ER into mitochondria where adenosine triphosphate (ATP) production is facilitated [12]. Recently, it has been proposed that σ_1_ receptors affect store-operated Ca^2+^ Entry (SOCE), with agonists, such as (**+**)-**pentazocine**, increasing the SOCE activity [13].

The σ_1_ subtype is also able to protect against ER stress associated with reactive oxygen species (ROS), which can easily activate the inositol-requiring enzyme 1 (IRE1) at MAM. In this context, the σ_1_ receptor stabilizes IRE1 and enhances cellular survival, prolonging the activation of the IRE1–XBP1 signaling pathway [14]. Moreover, the interaction of σ_1_ with B-cell lymphoma 2 (Bcl-2) or nuclear factor erythroid 2-related factor 2 (Nrf2) pathways is able to protect against apoptosis [15]. It is now clear that the σ_1_ receptor, which is defined as a ‘pluripotent chaperone’ [16], interacts with a number of proteins, as recently reviewed by Schmidt and colleagues [17], although more evidence is required to ascertain a functional interaction.

This pluripotent nature of the σ_1_ receptor is responsible for a series of complex effects with the cell state, such as stress conditions, which can produce modifications in the σ_1_-mediated response to stimuli. As recently reviewed, it is likely through these protein–protein interactions that the σ_1_ receptor exerts its action in viral infections and also against SARS-CoV-2 [18]. It has been demonstrated that the σ_1_ receptor interacts with the NSP6 viral protein, and its knock-out (KO) and knock-down (KD) abate the cells’ infection by both SARS-CoV-1 and 2, prompting focused research in this direction. By contrast, the KO and KD of the σ_2_ subtype, which interact with the viral protein ORf9c, do not impact on cells’ infection [19]. 

The neuroprotective action of σ_1_ receptor agonists is well established, and it has been proved that this activity is exerted through different mechanisms such as intracellular Ca^2+^ regulation, the prevention of oxidative stress, and anti-apoptotic effects. The σ_1_ subtype contributes to protein homeostasis: it can stimulate neurotrophin receptor signaling and reduce protein aggregation responsible for neurodegenerative disease, and it can also activate autophagy as a protective mechanism against damage arisen by misfolded proteins. Recently, many studies proved the ability of σ_1_ receptors to directly or indirectly interact with receptors or enzymes with key roles in neurodegeneration, particularly in Alzheimer’s disease (AD). Importantly, in patients with AD, a reduction of σ_1_ receptor density [20] has been demonstrated. Some studies attributed such reduction to the E4 variant of the apolipoprotein E gene (APOE 4) [21], although some controversies still exist [22]. All these mechanisms can promote cell survival and consolidate the role of the σ_1_ receptor as a target for therapies against neurodegeneration [23].

From a structural point of view, the σ_1_ protein has a 223 amino acid sequence, conserved among vertebrates, but devoid of similarity with any other protein. The closest protein seems to be ERG2p, which is a C8-C7 sterol isomerase expressed in the yeast. The two proteins share the same steroid binding domain-like (SBDL) regions, although no isomerase activity has been shown for the σ_1_ receptor [24]. A breakthrough milestone in the σ_1_ receptor-related research is the crystal structure of the protein, which was published in 2016 [25]. Later, also the crystal structures of the σ_1_ protein with agonists and antagonists bound to it were disclosed [26], and important insights into the binding modes of the σ_1_ ligands were provided with evidence for the so far ligands-based generated models. The most important interaction between ligands and the receptor is represented by an electrostatic bond between a basic amine (present in all the high-affinity σ_1_ ligands) and Glu172, while the rest of the ligand is accommodated in two different hydrophobic areas, which is in line with Glennon’s pharmacophore model [27]. 

According to this model, the σ_1_ receptor pharmacophore consists of a basic amine site tolerating a little substituent (which could be H or small alkyl groups) and two hydrophobic regions: the *Primary Hydrophobic Region* is 6–10 Å far from the amine site, while the *Secondary Hydrophobic* one is 2.5–3.9 Å far from the amine. Such a model very well fits into the crystal structure, whose availability has rationalized the binding of several molecules to σ_1_ receptors [28].

Importantly, crystallization revealed a trimeric complex, suggesting that the protein acts through polymerization states. Each subunit is formed by a single transmembrane domain (TMD) and a large cytosolic region that contains the binding pocket for each monomer. The 223 amino acid sequence is organized into five α-helices and ten β-strands: the *N*-terminus passes through the membrane, forms the only TMD, and protrudes in the ER lumen. The *C*-terminus is a flat hydrophobic sequence associated with the cytosolic surface of the ER. Within the binding pocket, besides the ionic interaction with Glu172, σ_1_ receptor ligands interact with Tyr103 (through hydrogen bonds with hydrophilic residues), which provides a favorable environment for the hydrophobic bonds within the primary hydrophobic region (formed by Val84, Ala86, Trp89, Met93, Leu95, Ala98, Tyr103, Leu105, Phe107, Tyr120, Ile124, Trp164, Met170, Ile178, Thr181, Leu182, Phe184, Ala185, Thr202, and Tyr206). The secondary hydrophobic region (made of Trp89, Ser117, Tyr120, Ile124, Asp126, Phe133, Val152, His154, Thr160, Val162, Trp164, and Met170, with Ser117 and His154 exhibiting their hydrophobic side chain to the cavity) tolerates smaller groups compared to the primary one [27,28]. Interestingly, the crystal structure of the receptor bound to its ligands revealed how the agonists, in contrast to the antagonists, induce a structural shift of the α 4 helix, likely preventing the oligomerization state, supporting that the action of the σ_1_ receptor occurs through protein–protein interaction (PPI). Therefore, while σ_1_ ligands may function as allosteric modulators of PPIs, the different oligomeric structures could be responsible for the many activities performed by these versatile receptors, with agonists and antagonists differently influencing the association among protomers: agonists produce lower oligomeric structures such as monomers and dimers, while antagonists produce higher ones [29].

### Overview on σ_1_ Receptors Ligands: Functional Activity, Proposed Endogenous Ligands, and Reference Compounds

*Functional activity.* Although the terms *agonist* and *antagonist* have been used to classify σ_1_ receptor ligands, an unambiguous definition of the functional activity (agonist versus antagonist) is often hard. As a matter of fact, both agonists (i.e., **fluvoxamine** and other selective serotonin reuptake inhibitors (SSRIs) [30]) and antagonists (i.e., **rimcazole** [31,32]) are effective in treating some pathologies such as psychosis, and there are some ligands (i.e., **BMY 14802** [33,34]) that show both agonist and antagonist activities in different conditions [35].

With the aim to unequivocally ascertain agonist vs. antagonist activity, many studies have been carried out. Among them, an interesting approach was developed by Gomez-Soler and colleagues, who developed a biosensor exploiting the Fluorescence Resonance Energy Transfer (FRET) technique in living cells. The model was based on the genetic decoration of the σ_1_ receptor with cyan and yellow fluorescent proteins. The distance between these two portions depends on the receptor conformation, which is influenced by the nature of the ligand. In fact, while agonists make the two fluorescent portions more distant, antagonists produce a conformational change that makes the portions closer, resulting in a fluorescence emission due to the energy transfer between them [36]. Therefore, the claimed σ_1_ receptor agonists and antagonists studied produced decreased and increased FRET signals, respectively. Another step in the same direction was taken by Yano et al., who produced a novel receptor homomer assay based on the Bioluminescence Resonance Energy Transfer (BRET) technique. The σ_1_ receptor was fused in its C-terminal and N-terminal sites with NanoLuciferace (NL) (BRET donor) and the yellow fluorescent protein Venus (VN), which works as an acceptor. The most suitable fusion construct pairs were identified to evaluate the ligand-induced BRET signal, and antagonists were shown to promote higher-order homomerization [35], with results from this assay in agreement with biochemical assays. The same BRET approach was also used to evaluate the heteromerization between the σ_1_ receptor and BiP, with the former protein bearing NL and the latter bearing the VN portions. Again, **haloperidol** and (**+**)-**pentazocine** displayed an opposite trend. Nevertheless, the same authors did not use the definition ‘agonist’ or ‘antagonist’ but preferred to discuss the phenotypes of the studied molecules, as some unexpected discrepancies emerged in the ligands studied (e.g., NE100, cocaine). All in all, even if a clear distinction between the two classes is still missing, these fluorescent-based assays provide useful information about the conformational changes that underlie σ_1_ receptors’ mechanisms of action.

*Proposed endogenous ligands.* The σ_1_ protein has been considered an orphan receptor for a long time because of the lack of an endogenous ligand. Nevertheless, some years ago, the endogenous hallucinogen compound *N,N*-dimethyltryptamine (**DMT**) showed to protect σ_1_ receptors by photolabeling performed using two radioactive photoaffinity labels: 3-[^125^I]iodo-4-azidococaine ([^125^I]-IACoc), more specific for σ_1_ receptors and 1-*N*-(2′,6′-dimethyl-morpholino)-3-(4-azido-3-[^125^I]iodo-phenyl)propane ([^125^I]IAF), with affinity for both σ receptor subtypes. **DMT** provided a dose-dependent protection when either [^125^I]-IACoc or [^125^I]IAF was used. Moreover, **DMT** showed to inhibit voltage-gated Na^+^ ion channels in both native cardiac myocytes and heterologous cells that express σ_1_ receptors. This pharmacological profile suggested **DMT** as an endogenous agonist for the σ_1_ receptor (*K*_d_ = 14.75 μM) [37]. In addition to **DMT**, **Progesterone**, which binds both σ subtypes (σ_1_ *K*_i_ = 239 nM, σ_2_ *K*_i_ = 441 nM [38]), has been also proposed as an endogenous σ_1_ receptor antagonist able to coordinate endocrine, immune, and CNS [38,39]. This is not surprising if we consider that σ_1_ receptors share homology with yeast sterol isomerase [24]. However, these ligands display low σ_1_ receptor affinity, and their blood concentration is too low for these ligands to be considered endogenously active. Plenty of other ligands can bind σ_1_ receptors with high affinity. Molecular dynamic simulations at the σ_1_ receptor binding pocket have shown how the diverse chemical structures to which high-affinity σ_1_ receptor ligands belong may easily be accommodated producing different associations among protomers that lead to the different activities observed.

Reference compounds. The most important classes of ligands are briefly summarized below (Table 1):

(*+*)-*Benzomorphans as agonists*: This class of ligands has a great historical value, as (+)-*N*-allylnormetazocine ((**+**)-**SKF**-**10047**) led to distinguishing a new group of receptors different from opioid receptors [40] that were named σ, after the first letter of the compound code [41]. Moreover, (**+**)-**Pentazocine** is still a σ_1_ reference compound, and its tritium radiolabeled form, which shed light on the existence of two classes of σ receptors [4], is still used for σ_1_ receptor radioligand binding assays.
*Antipsychotics as antagonists*: **Haloperidol** has high affinity for σ_1_ receptors (σ_1_ *K*_i_ = 8 nM [9]), but the involvement of the σ_1_ protein in schizophrenia has not completely been demonstrated. Several investigations on σ_1_ receptor gene polymorphs in this context are underway [42,43].
*Antidepressants as agonists*: **Imipramine**, **Fluoxetine**, **Fluvoxamine**, and **Sertraline** showed moderate to high affinities for σ_1_ receptors (*K*_i_ values ranging from 30 to 300 nM) [44], and several σ_1_ receptor agonists have shown antidepressant-like effects in animal models of depression, proving the involvement of the protein in depression [45,46].
*Neurosteroids as agonists/antagonists*: This class contains both agonists and antagonists. **Progesterone** in fact is a σ_1_ receptor antagonist, while **deoxycorticosterone**, **testosterone**, **pregnenolone–sulfate**, and **dehydroepiandrosterone** (**DHEA**) can produce agonist activities. All these ligands have unconventional structures because of the absence of a basic nitrogen that determines weak affinities for σ_1_ receptors. Neurosteroids have been proposed as endogenous ligands for σ_1_ receptors, as their effect seems also to be mediated by σ_1_ receptor binding [39,47].
*Others:* A number of other ligands have been synthesized with the aim to better understand the role of these receptors in different pathologies. The most representative examples are as follows:
  - **SA4503**: a highly selective agonist proved to exert effects on memory, depression, cardiac hypertrophy, and hearing [48,49,50,51,52];
  - **PB190**: a selective agonist that showed neuroprotective and antidepressant activities [53,54,55];
  - **PRE-084**: a selective agonist with beneficial effects on learning impairment [56,57,58];
  - **NE-100**: a σ_1_ reference antagonist often used to counteract the action of claimed σ_1_ agonists [59,60];
  - **PB212**: a subnanomolar affinity and selective σ_1_ antagonist [53,61];
  - **AC915**: a highly selective ligand with an antagonist profile [62];
  - **Pridopidine**: initially considered a dopaminergic D_2_ receptor antagonist, it was later repositioned as a selective σ_1_ receptor agonist (see Section 3.5) [63];
  - **FTC-146**: the ligand with the highest affinity and selectivity for σ_1_ receptor. It has been radiofluorinated and employed in Positron Emission Tomography (PET) in mice, rats, squirrels, monkeys, and humans [64,65,66];
  - **S1RA**: a σ_1_ receptor antagonist developed by Esteve company for the treatment of neuropathic pain and the strengthening of opioid analgesia, which successfully completed Phase I clinical trial showing safety and tolerability [67,68];
  - **BD1063**: a σ_1_ receptor antagonist with anti-cocaine effects in mice [69,70].

**Table 1:** Representative σ_1_ receptor (σ_1_ R) ligands and their affinities for σ_1_ R and σ_2_ receptor (σ_2_ R).

Name	Structure	σ_1_ R *K*_i_ nM	σ_2_ R *K*_i_ nM	Reference for Binding Values
**(+)-Benzomorphans:**				
**(+)-SKF-10047**	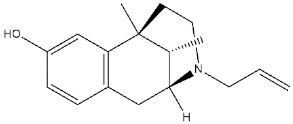	153	154335	[9]
**(+)-pentazocine**	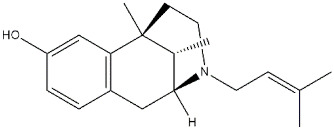	15.4	3475	[9]
**Antipsychotics:**				
**Haloperidol**	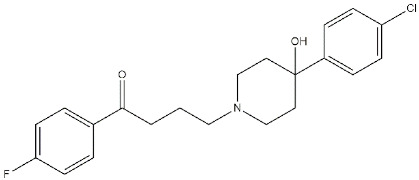	1.09	41.9	(σ_1_ R) [71] (σ_2_ R) [9]
**Antidepressants:**				
**Imipramine**	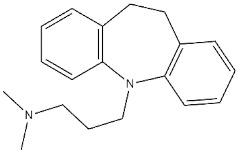	343	2107	[44]
**Fluoxetine**	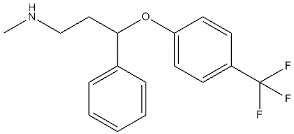	240	16100	[44]
**Fluvoxamine**	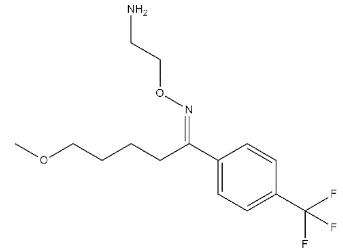	36	8439	[44]
**Sertraline**	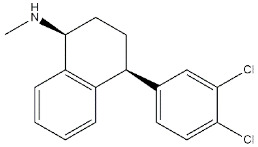	57	5297	[44]
**Neurosteroids:**				
**Progesterone**	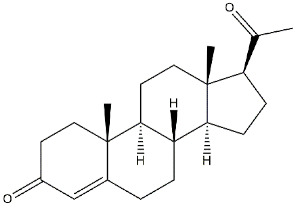	239	441	[38]
**Deoxycorticosterone**	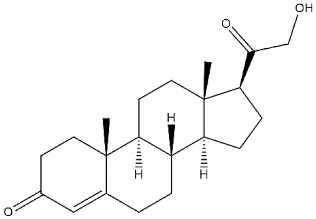	938	n.d.	[39]
**Testosterone**	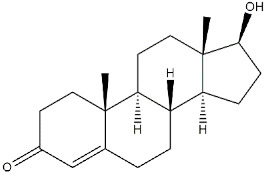	1014	n.d.	[39]
**Pregnenolone-sulphate**	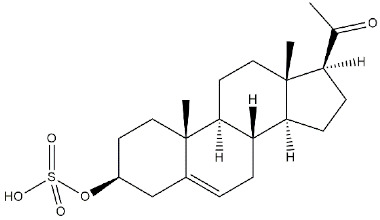	3196	n.d.	[39]
**DHEA**	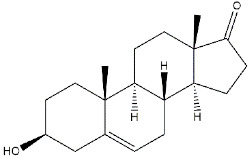	2.959	n.d.	[47]
**Others:**				
**SA4503**	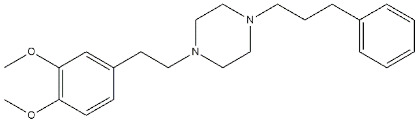	4.63	63.09	[72]
**PB190**	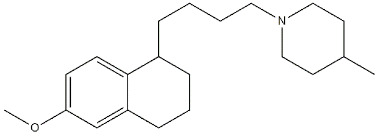	0.42	36.3	[53]
**PRE-084**	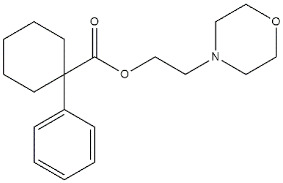	53.2	32.100	[73]
**NE-100**	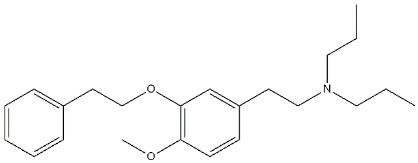	1.1	170	[74]
**PB212**	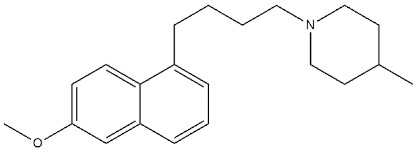	0.030	17.9	[53]
**AC915**	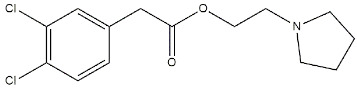	4.89	>10000	[62]
**Pridopidine**	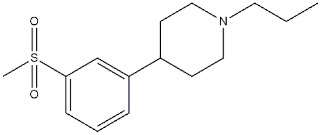	57	5450	[75]
**FTC-146**	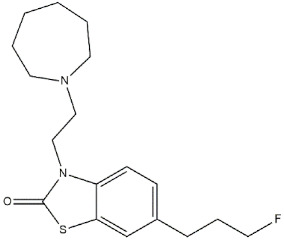	0.0025	364	[64]
**S1RA**	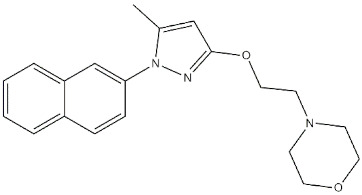	23.5	>1000	[76]
**BD1063**	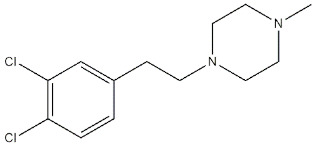	9.15	449	[70]

## 3. σ_1_ Receptor Ligands in a MTDLs Approach

In addition to the direct neuroprotective action of σ_1_ receptors, the additional modulation of targets such as opioid, N-methyl-D-aspartate (NMDA), dopaminergic, and cholinergic receptors renders the σ_1_ protein an intriguing target for the modulation of processes that involve these systems, prompting the development of σ_1_-based MTDLs for the treatment of complex and multifactorial pathologies such as AD. 

### 3.1. MTDLs Acting at σ_1_ Receptor and Cholinergic System

Drug metabolism leads to metabolites that can be more potent or less potent than the parent drug. In the former case, the drug can be considered a sort of prodrug. Thus, the simultaneous presence of the two chemical entities may produce a synergistic powerful effect.

Starting from this idea, in 2010, Lecanu et al. developed pairs of drugs and the corresponding prodrugs, which act on different targets such as acetylcholinesterase (AChE) whose inhibition is a consolidated therapeutic strategy in AD, and σ_1_ receptors. With this aim, two series of molecules were built: hydroxyphenyl-butan-1-ones (drug) and their corresponding carbamates (prodrugs). Upon inhibition of the AChE, the prodrug releases the drug. Thus, the prodrug and drug bind and act through the σ_1_ receptor and AChE, while the drug exerts its antioxidant action. 

**Figure 1:**
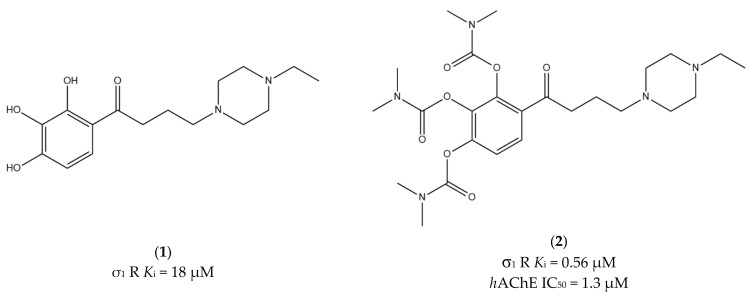
Compound (**1**) and (**2**) and their *K*_i_ and IC_50_ values for σ_1_ receptor (σ_1_ R) and human acetylcholinesterase (*h*AChE) [77].

Among the studied ligands, two compounds (i.e., 2,3,4-trihydroxy-phenyl)butan-1-one **1** and the corresponding *N*,*N*-dimethylcarbamoyloxy prodrug **2**; Figure 1) showed the best compromise in terms of activity on the different targets. Compound **2** was able to (i) inhibit AChE with a potency comparable to reference compounds; (ii) bind σ_1_ receptors (high selectivity, sub-micromolar affinity, and antagonist activity); and (iii) prevent mitochondrial toxicity by inhibiting mitochondrial complex IV and V. On the other hand, compound **1**, even if much less active than its bio-precursor toward σ_1_ receptors, was also able to attenuate reactive oxygen species (ROS), Aβ_1-42_-induced neurotoxicity, and mitochondrial toxicity by inhibiting complexes I, II, IV, and V.

Moreover, **2** was demonstrated to cross the blood–brain barrier (BBB) very shortly after its administration and be transformed into **1** very quickly.

Behavioral studies on animal models are needed, but preliminary data suggest that this multi-target profile can represent a novel therapeutic strategy to treat AD [77].

**Figure 2:**
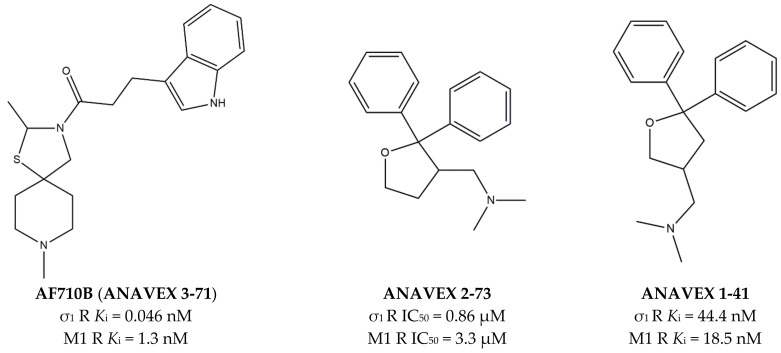
**ANAVEX 3-71**, **2-73** and **1-41** structures and their *K*_i_ and IC_50_ values for σ_1_ receptor (σ_1_ R) and muscarinic M1 receptor (M1 R) [82,84,85].

Many pharmacological and genetic data have proven that activation of muscarinic M1 receptors (mAChRs) attenuates symptoms of neurodegenerative pathologies [78,79], as in the case of MTDLs able to bind both M1 and σ_1_ receptors. The most investigated compound representative of this class is **ANAVEX 2-73** (also known as **Blarcamesine**) (Figure 2). This compound is in advanced clinical phases for several CNS diseases such as AD, Parkinson’s disease (PD), Rett, and Fragile X Syndromes (anavex.com/#!/pipeline, accessed Apr 3, 2021). In addition to binding M1 and σ_1_ receptors, **Blarcamesine** also binds M2–M4 receptors (with micromolar affinity), Na^+^ channel site 2, and NMDA receptor (NMDAR) [80,81]. In this context, Fisher and co-workers, who previously developed orthosteric M1 receptors agonists (e.g., **AF102B**, **AF267B,** and **AF292**), extended their approach in order to target also σ_1_ receptors. With this aim, compound **AF710B** (also known as **ANAVEX 3-71,** Figure 2) that can activate both M1 and σ_1_ receptors with high potency and selectivity was identified [66]. **AF710B** is a positive allosteric modulator (PAM) of M1 receptor, as it improves the efficacy of carbachol, and this activity, together with a comparable agonism at the σ_1_ receptor can preserve synaptic elements in vitro. In vivo studies performed on trihexyphenidyl-treated rats and 3xTg-AD mice showed that **AF710B** can restore cognitive deficits and attenuate signs of AD phenotypes by the reduction of β-secretase 1 (BACE1) levels, GSK3β and CDK5/p25 activity (which contribute to the hyperphosphorylation of tau protein), neuroinflammation, soluble and insoluble Aβ_40_ and Aβ_42_ plaques, and tau pathology [82]. In fact, **AF710B** reduces the expression of the putative BACE1, so that proteolytic fragments produced by β-secretase were considerably lower in 3xTg-AD treated than in untreated mice. Moreover, tau kinases GSK3β and CDK5 take part in the mechanism of hyperphosphorylation of tau protein. In particular, in AD patients, CDK5 activator p35 is cleaved to produce the protein p25, which binds with high affinity and activates GSK3β [83]. The activation of M1 receptors produces a reduction of GSK3β expression, while the activation of σ_1_ prevents the formation of CDK5/p25. Therefore, the inhibition of GSK3β and CDK/p25 by **AF710B**, upon interaction with both M1 and σ_1_ receptors, results as a promising approach in the treatment of tauopathies [82].

The effect of σ_1_ receptors in inflammation through microglia modulation has been reported [54,86,87], and **AF710B** was shown to reduce reactive astrocytes and activated microglia in the animals, as detected by the low levels of glial fibrillary acidic protein (GFAP) and ionized calcium-binding adapter molecule 1 (Iba-1). Notably, astrocytes and microglia are increased in number and size in AD patients.

Another study performed using **AF710B** on McGill-R-Thyl-APP transgenic (tg) rats revealed that this MTDL can reduce amyloid pathology and markers of neuroinflammation while increasing amyloid cerebrospinal fluid clearance and synaptic marker. The most important achievement is represented by the prolonged duration of these effects, which are maintained five weeks after the treatment is interrupted [88].

In addition to **AF710B** and **ANAVEX 2-73,** Anavex Life Sciences Corp portfolio comprehends an isomer of **ANAVEX 2-73**, named **ANAVEX 1-41** (Figure 2) that next to the activity toward σ_1_ and M1 receptors, also displays activity for α_1_, 5-HT_2_, and D_3_ receptors [81], with an indication for the treatment of depression, stroke, and neurodegenerative diseases (anavex.com/#!/pipeline).

### 3.2. MTDLs Acting at σ_1_, 5-HT_4_ Receptors and AChE

**Figure 3:**
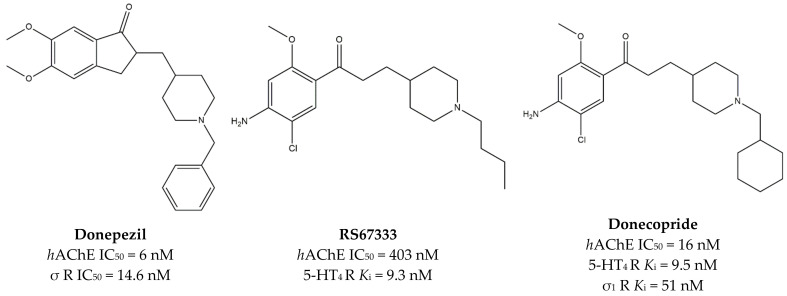
**Donepezil**, **RS67333**, and **Donecopride** structures and their *K*_i_ and IC_50_ values for σ receptors (σ R), σ_1_ receptor (σ_1_ R), human acetylcholinesterase (*h*AChE) and serotoninergic 5-HT_4_ receptor (5-HT_4_ R) [89,90,91].

In 2015, Rochais and co-workers developed the MTDL **Donecopride** from the combination of the AChE inhibitor **Donepezil** and the serotoninergic 5-HT_4_ receptor (5-HT_4_R) agonist **RS67333** (Figure 3). Indeed, **Donecopride** inhibits AChE and exerts a partial 5-HT_4_R agonist activity on 5-HT_4_R.

Subsequent studies revealed that **Donecopride** has high σ_1_ receptor affinity (*K*_i_ = 51 nM [90]), which is in agreement with the nanomolar affinity of **Donepezil** for the same target [91].

This finding inspired many studies about the possible therapeutic exploitation of such combinations of activities [92,93,94,95]. Starting from these pieces of evidence, Lalut et al. postulated that the replacement of the benzene ring by an indole group, together with the insertion of a piperidine-chained spacer, could improve affinity for the σ_1_ receptor. Thus, *N*-substituted and *N-*unsubstituted indoles were developed, with diverse decoration on the indole and piperidine rings.

The *N-*unsubstituted indoles displayed a profile comparable to their lead compound in terms of affinity at 5-HT_4_ receptors and inhibitory activity at AChE. The most promising ligands were tested in vitro on Jukart cell membranes for the determination of the σ_1_ affinity, according to Ganapathy [96].

**Figure 4:**
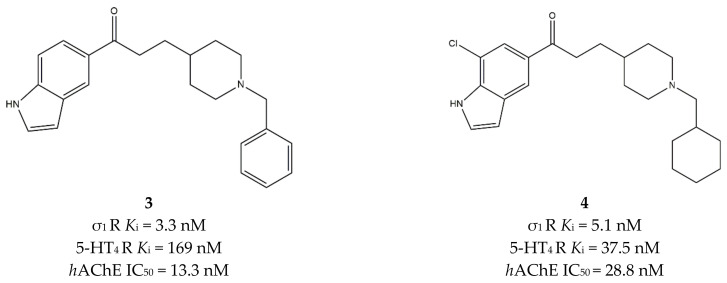
Compounds (**3**) and (**4**) structures and their *K*_i_ and IC_50_ values σ_1_ receptor (σ_1_ R), serotoninergic 5-HT_4_ receptor (5-HT_4_ R) and human acetylcholinesterase (*h*AChE) [90].

The *N*-benzyl derivative **3** (Figure 4) displayed the highest affinity for the σ_1_ receptor and the most potent inhibition toward AChE but a moderate affinity for 5-HT_4_ receptors. The best compromise among affinities at σ_1_ and 5-HT_4_ receptors and activity at AChE was reached by the cyclohexylmethyl derivative **4** (Figure 4), which was thus considered the lead compound of the study.

This compound was tested in vivo using the dizocilpine-induced amnesia assay in mice. In the 0.1–1 mg/kg dose-range, **4** did not attenuate the dizocilpine-induced spontaneous alternation performance deficit, while at lower doses, the passive avoidance deficit was attenuated with a 40% protection. The authors concluded that the involvement of the σ_1_ receptor needed further explorations by including challenges with the σ_1_ receptor antagonist **NE-100** [90].

### 3.3. MTDLs Acting at σ_1_ Receptor, AChE, BuChE, BACE1, MAO-A, MAO-B, 5-LOX, ROS, and Stem Cells

Another important class of MTDLs able to treat AD is represented by ligands developed by Estrada and co-workers in 2016. Their strategy was based on the synthesis of MTDLs able to inhibit AChE/Butyrylcholinesterase (BuChE), BACE1, and bind σ_1_ receptors.

For the study, the authors combined lipoic acid (LA), whose antioxidant properties [97] are exploited in AD [98], together with either the *N*-benzylpiperidine (NBP) moiety (featured by **Donepezil**, as discussed above) or the *N,N*-dibenzyl-*N*-methylamine (DBMA) moiety of the BACE1 inhibitor **AP2238**. This approach led to the MTDLs LA-NBPs and LA-DBMA, respectively.

More than fourteen ligands were developed, and some of them were tested for σ receptors affinity. *K*_i_ values in the sub-micromolar and sub-nanomolar range were found for σ_1_ receptors, and radical scavenging properties were evaluated using the Oxygen Radical Absorbance Capacity (ORAC) assay, with LA-based ligands showing good antioxidant capacities, comparable to vitamin E.

**Figure 5:**
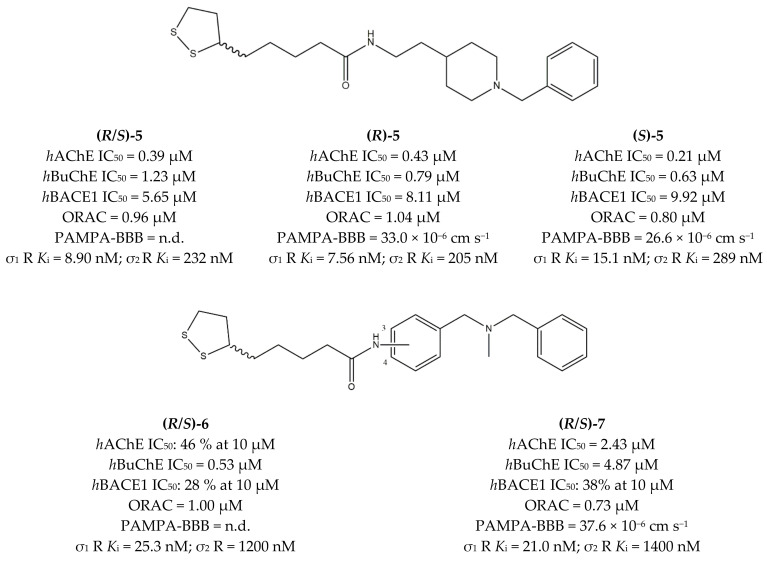
Compounds (**5**,**6**) and (**7**) structures and pharmacological profile for σ receptor (σ_1_ R and σ_2_ R), human acetylcholinesterase (*h*AChE), human butyrylcholinesterase (*h*BuChE), β-secretase 1 (BACE1), Oxygen Radical Absorbance Capacity (ORAC) and parallel artificial membrane assays (PAMPA-BBB) [99].

In the LA-NBP class, the lowest *K*_i_ value at the σ_1_ receptor was displayed by ligand **(*R*)-5**; in the LA-DBMA series, the best σ_1_ receptor affinity values were displayed by enantiomeric mixtures of compounds **6** and **7**, which differ for the position of the amidic substituent on the central aromatic ring (3-position for compound **6** and 4-position for compound **7**) (Figure 5). Both compounds showed *K*_i_ values in the nanomolar range, with no enantioselectivity.

In addition, compounds **5** and **7** showed promising in vitro absorption, distribution, metabolism, excretion and toxicity (ADMET) properties, as they are able to cross the BBB by in vitro parallel artificial membrane assays (PAMPA-BBB). They also displayed low or moderate cytotoxicity in the SH-SY5Y neuroblastoma cell line (supporting their s_1_ agonist or partial agonist rather than antagonist activity) [99].

In order to develop a more holistic strategy to hit AD, the same group designed novel MTDLs to target monoamine oxidase A (MAO-A), monoamine oxidase B (MAO-B), lipoxygenase-5 (5-LOX), AChE/BuChE, and σ_1_ receptors. Additionally, ROS attenuation and mechanisms of differentiation of neural stem cells were taken into account for these novel compounds. Thus, twenty-nine new Donepezil-flavonoid hybrids (DFHs) were synthesized. The *N*-benzylpiperidine fragment was connected through an amide or ester linker to the 4-oxo-4*H*-chromene flavonoid scaffold, which is endowed with MAO-A, MAO-B, and 5-LOX inhibitory activity, as well as antioxidant, anti-inflammatory, and neurogenic properties. Moreover, the flavonoid scaffold underwent some modifications such as substitution of the 4-oxo-4*H*-chromene with a quinoline-4(1*H*)-one or quinoline and the introduction of hydroxyl/methoxy groups on the aromatic ring.

**Figure 6:**
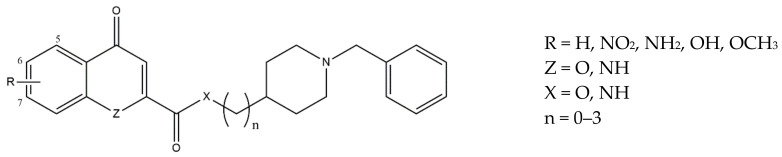
General structures of 4-chromenone and 4-quinolone classes of ligands.

Three classes of compounds (i.e., *4-chromenone*, *4-quinolone*, and *quinoline-2-carboxamide*) were tested for their activity at AChE/BuChE, 5-LOX, and MAOs as well as for their BBB permeability and antioxidant capacities. A restricted group of ligands belonging to the *4-chromenone* and *4-quinolone* classes (general structure in Figure 6) was selected and tested at σ receptors, showing low nanomolar *K*_i_ values (0.98 nM > *K*_i_ > 47.4 nM) and selectivity versus the σ_2_ subtype. The best affinities were obtained when a single hydrogen-bond acceptor group (NO_2_ > NH_2_ > OH > OCH_3_) was in the 6-position of either the 4-oxo-chromene or 4-oxo-quinoline system, while the length of the linker between the amide and the *N*-benzylpiperidine groups had no influence on the affinity for σ_1_ receptors.

The best compromise among all studied compounds is represented by compound **8** (R = 6,7-diOMe; Z = O; X = NH and n = 2, Figure 6), which showed good neuroprotective properties: *h*AChE IC_50_ = 46 nM; *h*BuChE IC_50_ >10000 nM; PAMPA-BBB = 5.9 × 10^−6^ cm s^−1^; 5-LOX IC_50_ = 74.3 µM; MAO-A IC_50_ = 15.3 µM; MAO-B IC_50_ = 5.2 µM; ORAC = n.a.; σ_1_ R *K*_i_ = 37.4 nM; σ_2_ R *K*_i_ = 239 nM.

Moreover, compound **8** was shown to promote the differentiation of adult mice neural stem cells (NSC), and its predicted metabolites have been analyzed using the Derek Nexus program and shown to be not toxic. Additionally, docking by Molecular Dynamics (MD) simulation into AChE, 5-LOX, and the σ_1_ receptor showed low ΔG_bind_ values in agreement with the experimentally detected interactions with the three targets. Based on these data, compound **8** was proposed as a promising candidate against AD and neurodegenerative diseases in general [100]. 

**Figure 7:**
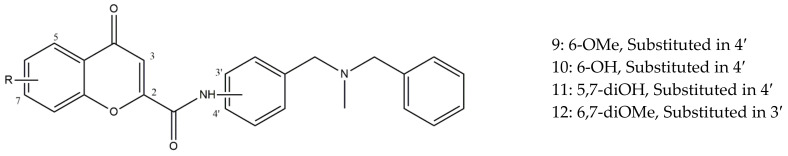
General structure of 4-chromenone-*N,N*-dibenzyl-*N*-methylamine (DBMA) derivatives.

Based on these encouraging data, the same group developed other MTDLs targeting AChE/BuChE, BACE1, MAOs, 5-LOX, the differentiation of neural stem cells, and σ_1_ receptors. With this aim, the already exploited flavonoid cores (i.e., 4-chromenone or 4-quinolone) were conjugated with the *N,N*-dibenzyl-*N*-methylamine (DBMA) fragment, which was chosen for its proven interaction with the catalytic anionic site (CAS) of AChE. The two classes of *4-chromenone* and *4-quinolone* derivatives were tested, and the best compromise was obtained with ligands belonging to the former class. The most promising ligands (i.e., **9**, **10**, **11**, and **12**, Figure 7) were also tested at σ_1_ and σ_2_ receptors, showing comparable sub-micromolar affinities for σ_1_ and micromolar affinities for σ_2_ receptors. These results showed that functionalization in 3′ or 4′ of the central benzene ring as well as changes in the position of the methoxy/hydroxy groups on the 4-chromenone scaffold poorly influence the σ receptors’ affinity.

Despite its low affinity and selectivity for σ_1_ receptors (σ_1_ R *K*_i_ = 0.53 µM; σ_2_ R *K*_i_ = 1.30 µM), compound **12** represented the best compromise in terms of activity toward different targets (*h*AChE IC_50_ = 4.5 µM; *h*MAO-A IC_50_ > 100 µM; *h*MAO-B IC_50_ > 100 µM; 5-LOX IC_50_ = 30.4 µM; and PAMPA-BBB = 18.8 × 10^−6^ cm s^−1^). ΔG_bind_ values obtained through docking to AChE, 5-LOX, BACE1, and σ_1_ were in agreement with the experimental ones, while a relatively safe profile was predicted in terms of toxicity. However, a low-confidence alert (below 67%) for human ether-a-go-go-related gene (*h*ERG) channel inhibition was recorded, so that cardiac toxicity might be expected with this compound, which needs to be ascertained with further experimental studies. Neurogenic evaluation was performed on a primary culture of neural stem cells from the SGZ of adult rats, and compound **12** stimulated differentiation. Altogether, these data suggest compound **12** as a possible therapeutic agent that promotes brain auto-repair processes and blocks early steps of neurodegenerative cascades [101].

### 3.4. MTDLs Acting at σ_1_, NMDA Receptors, and VGCC

NMDA receptors are glutamate-gated Ca^2+^ permeable ion channels associated with σ_1_ receptors. In 1987, Quirion et al. identified the σ_1_ receptor as the phencyclidine (PCP) binding site [102], but further studies performed by the same group refuted the thesis and classified σ receptors as a separate class [103]. However, a strong connection between these two receptors has been found in the following years. σ_1_ Receptors can enhance Ca^2+^ influx via NMDAR, but they can also prevent toxicity induced by the excessive flux of this cation. This activity occurs through the modulation of NMDAR-associated intracellular signaling component, i.e., the neuronal Nitric Oxide Synthase (nNOS), which produces nitric oxide (NO) [104].

Low doses (100 nM) of σ_1_ receptor agonists enhance NMDAR function, producing a potentiation of the NMDAR response. Curiously, high doses (≥10 μM) do not exert a stronger effect. Indeed, a bell-shaped dose–response trend, which is typical of the σ_1_ receptor agonists-mediated action, can be observed accounting for the majority of the effects of σ_1_ receptor agonists on NMDAR response [105,106]. A potential explanation of this effect is that σ_1_ receptor agonists can directly bind NMDARs and block channel conductance [107], but further studies are needed to solve the question. Moreover, the NMDAR increased activity is fast and prolonged [108], so that interactions also with other targets may be involved [104]. Even if these mechanisms have not been completely clarified, an interaction between the σ_1_ receptor and NMDAR exists. In particular, as recently reviewed, σ_1_ receptors can associate together with GluN1, Glun2a, and Glun2b subunits [17].

Taking into consideration all these aspects, dual drugs acting on σ_1_ and NMDA receptors may be a promising strategy to target neurodegenerative disorders.

**Figure 8:**
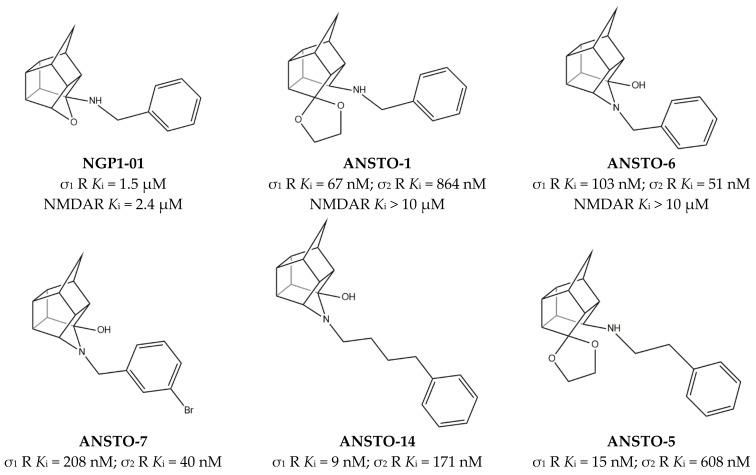
**NGP-01**, **ANSTO-1**, **ANSTO-6**, **ANSTO-14**, and **ANSTO-5** structures and their *K*_i_ values for σ receptor (σ_1_ R and σ_2_ R) and N-methyl-D-aspartate receptor (NMDAR) [110,111,112].

With the aim to discover potentialities of the aza-cage compound of the pentacyclo[5.4.0.0^2,6^.0^3,10^.0^5,9^]undecane ring system (PCU), Van der Schyf et al. engaged physiological studies using sheep Purkinje fibers. These studies classified **NGP1-01** (Figure 8) as the lead compound of a novel class of Ca^2+^ channel antagonist having a “cage” molecular structure [109].

Subsequent studies demonstrated the multi-target profile of **NGP1-01** evidenced by its ability to reduce Ca^2+^ influx through interaction with L-type voltage-gated calcium channel (VGCC) [113] and NMDAR, with an activity comparable to the NMDAR antagonist **memantine** [114].

**Figure 9:**
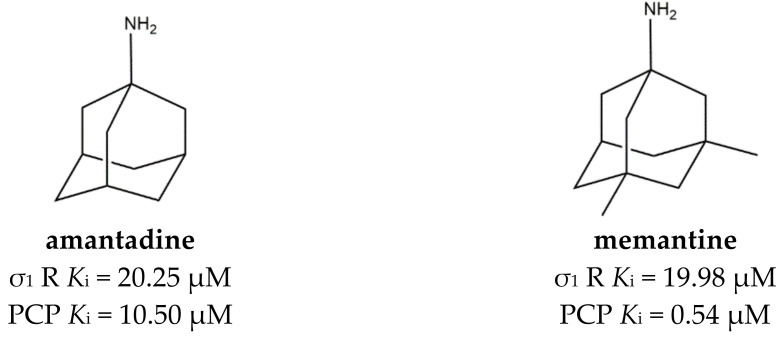
**Amantadine** and **memantine** structures and their *K*_i_ values for σ_1_ receptor (σ_1_ R) and the phencyclidine binding site at the N-methyl-D-aspartate receptor (PCP) [115].

The discovery that **amantadine** had micromolar *K*_i_ values for σ receptors [115] led to investigate whether cage compounds could bind σ receptors. The micromolar affinity for σ_1_ receptors of **NGP1-01** inspired the synthesis of new trishomocubanes pentacyclo[5.4.0.0^2,6^.0^3,10^.0^5,9^]undecylamines and 4-azahexacyclo[5.4.1.0^2,6^.0^3,10^.0^5,9^.0^8.11^]dodecanes, such as **ANSTO-1** and **ANSTO-6** (Figure 8). These compounds showed good affinity for σ receptors but low affinity for NMDAR (i.e., **ANSTO-1** and **ANSTO-6**, Figure 9) [110,111].

The subsequent SAR, 3D-QSAR, and docking studies clarified the following: (i) aza-trishomocubane derivatives have high affinity for both σ receptors; (ii) affinity for σ_1_ receptors is higher when the chain is longer; (iii) *para*- or *meta*-substitution with halogens is well tolerated, but the absence of substituents produces the highest selectivity and the lowest *K*_i_ value for σ_1_ receptors (**ANSTO-14**, **ANSTO-5**, and **ANSTO-7**, Figure 8) [103,104].

Nevertheless, the improved σ_1_ receptor affinity was accompanied by a dramatic loss in the affinity for NMDARs. Consequently, **NGP1-01** can still be considered the best MTDL in the class of the cage compounds.

Deeper studies able to clarify the mechanism of the σ_1_ receptors-mediated antineurodegenerative activity of **NGP1-01** are not available, to the best of our knowledge, but the affinity of the compound for the σ_1_ receptor and the antineurodegenerative properties mediated by the reduction of Ca^2+^ influx have been demonstrated, qualifying **NGP1-01** as a potential MTDL that is useful in the treatment of neurodegenerative pathologies.

Among cage compounds, **amantadine** and **memantine** (Figure 9) deserve to be mentioned because of their well-known antagonistic activity at the phencyclidine (PCP) binding site of NMDARs [116] together with the already mentioned affinity for σ_1_ receptors [115]. However, while the affinity of **memantine** at NMDARs is around 40-fold higher than the affinity at σ_1_, **amantadine** interacts to the same extent with σ_1_ and NMDARs. Additionally, Peeters et al. confirmed the agonist σ_1_ receptor profile and shed the light on **amantadine** ability to positively increase dopaminergic transmission through the activation of σ_1_ receptors [117]. Therefore, **amantadine** can be considered a full-fledged MTDL.

### 3.5. MTDLs Acting at σ_1_ Receptor/Dopaminergic Transmission

σ_1_ Receptors are also associated with the dopaminergic transmission. BRET, coimmunoprecipitation (Co-IP), and proximity ligation assays studies demonstrated that σ_1_ receptors interact with dopamine (DA) D_1_ and D_2_ receptors, as well as with dopamine transporter (DAT) [17]. Beneficial effects of the σ_1_ chaperone function on dopaminergic transmission may be exploited in pathologies such as AD, PD, Huntington’s disease (HD), and schizophrenia.

Interactions with D_1_ receptors were studied by Moreno et al. that performed in vitro and ex vivo assays, which led to identify, in the presence of **cocaine**, the formation of a heterotrimer composed of σ_1_, histamine H_3_, and D_1_ receptors. Upon the formation of this trimer, the inhibition of D_1_ receptors was abolished, and G_s_ proteins were activated with an increase of cAMP, recruitment of β-arrestin, and increase in p-ERK1/2 levels [118]. As a result, σ_1_ antagonists have the potential to reduce **cocaine** effects and consequent **cocaine** addiction.

σ_1_ Receptors can also form heteromers with the D_2_ receptor. Biophysical, biochemical, and cell biology assays performed on mouse striatum demonstrated that the administration of cocaine can induce the formation of σ_1_–D_2_ dimers, inhibiting the downstream signaling [119].

Finally, σ_1_ receptors are also able to associate with DAT and reduce DAT-mediated dopamine efflux produced by **methamphetamine** exposure [120].

A large number of dopaminergic agents have been examined to better define their pharmacological profiles, and some of them were found to bind σ_1_ receptors, in accordance with a partially overlapping pharmacophore.

**Figure 10:**
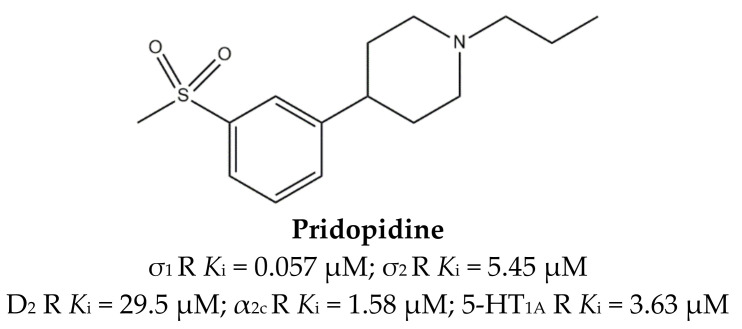
**Pridopidine** structure and its *K*_i_ values for σ (σ_1_ R and σ_2_ R), dopaminergic D_2_ (D_2_ R), alpha-2c adrenergic (α_2c_ R) and serotoninergic 5-HT_1a_ (5-HT_1a_ R) receptors [75].

For a long time, **Pridopidine** (Figure 10) has been considered a dopaminergic antagonist because of its micromolar affinity for D_2_ receptors, but some in vivo data and its structural resemblance to the σ_1_ receptor ligands inspired Sahlholm and co-workers to perform studies that defined **Pridopidine** as a highly selective σ_1_ receptor agonist [63,121].

To better understand the neuroprotective and neurorestorative role of **Pridopidine**, Francardo et al. assayed the drug in a mouse model of Parkinson’s disease (unilateral 6-hydroxydopamine (6-OHDA) lesion). After 5 weeks of daily administration of a low dose (0.3 mg/kg), **Pridopidine** produced an improvement in forelimb use and abolished the ipsilateral rotational bias typical of hemiparkinsonian animals. Moreover, the protection of nigral cell bodies, increased dopaminergic fiber density in the striatum, striatal upregulation of glial cell line-derived neurotrophic factor (GDNF), and brain-derived neurotrophic factor (BDNF) and phosphorylated extracellular signal-regulated kinases 1/2 (ERK1/2) were also observed [122]. At higher doses, **Pridopidine** was able to interact with other targets such as alpha-2 adrenergic α_2C_ and serotonin 5-HT_1A_ receptors, providing a solution to reduce L-dopa-induced dyskinesia (LID), both in PD and HD [75]. Of note, a phase III trial is in progress to evaluate the efficacy and safety of **Pridopidine** in HD patients (PRidopidine’s Outcome on Function in Huntington Disease, PROOF-HD, ClinicalTrials.gov Identifier: NCT04556656). Importantly, promising data of **Pridopidine** for the treatment of AD are emerging as the compound prevents mushroom spine loss in hippocampal cultures from APP knock-in (APP-KI) and presenilin-1-M146 V knock-in (PS1-KI) mice [123].

The ability to functionally interact with different receptors identifies **Pridopidine** as a MTDL, which is useful in the treatment of neurodegenerative disease.

**Figure 11:**
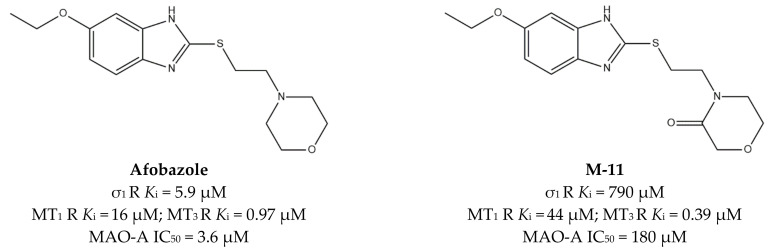
**Afobazole** and **M-11** structures and their *K*_i_ and IC_50_ values for σ_1_ receptor (σ_1_ R), melatonin MT_1_ and MT_3_ receptors (MT_1_ R and MT_3_ R) and monoamine oxidase A (MAO-A), [124].

**Afobazole** (Figure 11) is an anxiolytic drug designed and pharmacologically studied by FSBI “Research Zakusov Institute of Pharmacology”, Russia. This drug binds melatonin MT_1_ (*K*_i_ = 16 µM) and MT_3_ (*K*_i_ = 0.97 µM) receptors. MT_3_ is a regulatory site of quinone reductase 2 enzymes (NQO2), which is responsible for ROS production. For this reason, the reversible inhibition performed by **Afobazole** produces a cytoprotective effect.

Some studies performed on **Afobazole** metabolites revealed that one of them, M-11, can bind the MT_3_ receptor (*K*_i_ = 0.39 µM) with a slightly higher affinity, while a lower affinity is associated to the MT_1_ receptor (*K*_i_ = 44 µM). Consequently, **Afobazole** activity can be considered empowered by the additive activity of its metabolite **M-11** (Figure 11) [124].

**Afobazole** can also inhibit MAO-A, thus increasing adrenaline, noradrenaline, and dopamine levels in CNS. Additionally, it also behaves as a σ_1_ receptor regulator, producing a cytoprotective effect through the inhibition of ROS production [125].

The need to clarify the involvement of σ_1_ receptors in neurodegenerative diseases inspired some studies on the 6-OHDA-induced parkinsonism model in mice. The study revealed that the administration of **Afobazole** (2.5 mg/kg, *i.p.*) over 14 days was able to restore motor dysfunction as well as prevent decreases in dopamine in the 6-OHDA-lesioned striatum and the loss of tyrosine hydroxylase positive (TH+) neurons in the substantia nigra. Importantly, these activities were abolished by the administration of the σ_1_ receptor antagonist **BD-1047** (3.0 mg/kg, *i.p.*), demonstrating that the effects of **Afobazole** are mediated by the σ_1_ receptor [126].

These pieces of evidence prove a strict connection between the dopaminergic transmission and the σ_1_ receptor and encourage more in-depth investigation to clarify the mechanisms involved.

**Figure 12:**
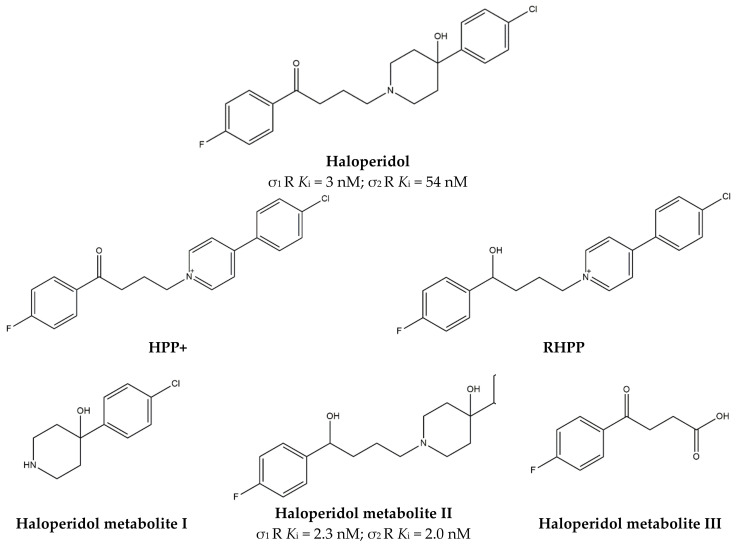
**Haloperidol** and its metabolites with *K*_i_ values for σ_1_ and σ_2_ receptors (σ_1_ R and σ_2_ R) [127,128,129].

With high affinity and antagonist properties at D_2_ receptors, **Haloperidol** (Figure 12) equally binds the σ_1_ receptor, behaving as an antagonist [127].

After administration, **Haloperidol** undergoes a complex metabolism that leads to toxic metabolites such as 4-(4-chlorophenyl)-1-[4-(4-fluorophenyl)-4-oxybutyl]pyridinium ion (HPP+) and 4-(4-(chlorophenyl)-1–4-(fluorophenyl)-4-hydroxybutyl-pyridinium (RHPP+) (Figure 12). In addition, also non-toxic metabolites have been observed (HP metabolites), such as HP metabolite II [130] and HP metabolites I and III [131] (Figure 12). Biological activities of the non-toxic HP metabolites (I, II, and III) were studied, and in particular, their affinities for σ_1_ receptors were evaluated. While HP metabolite III does not bind σ_1_ receptors, HP metabolite I exerts a reversible inhibition, and HP metabolite II exerts an irreversible inhibition at the σ_1_ receptor. Thus, haloperidol metabolites can reinforce its action [128].

Further studies are needed to better understand the exploitability of this pharmacological activity, but preliminary data suggest that σ_1_ receptor antagonists, such as **Haloperidol**, could be useful in blocking the acute toxicity and the rewarding effects of cocaine because of an influence on the DA release (pre-synaptic activity) and reduction of Ca^2+^ mobilization produced by the activation of D_1_ receptors (post-synaptic activity) [132]. The additional activity of **Haloperidol** and its active metabolites could be foreseen in the antinociceptive action occurring through the modulation of opioid receptors [133].

Moreover, studies performed by Marrazzo and colleagues revealed that HP metabolite II is also able to exert anticancer activity because of its antagonism at the σ_1_ receptor and agonism at the σ_2_ receptor [134]. A more detailed discussion of HP metabolite II anticancer potentialities is reported in Section 3.7.

### 3.6. MTDLs Acting at σ_1_ and μ-Opioid Receptors

One of the most difficult challenges in medicinal chemistry is represented by the synthesis of drugs able to manage pain for a prolonged period. In particular, chronic pain therapies are often associated with the adaptation phenomenon that is responsible for most of the side effects of these drugs, because it leads to periodic increases of the doses to be administered in order to keep the analgesia. Opioids, with their generally low therapeutic index, are still the most effective class of drugs able to relieve pain, making the need to increase the dose extremely life-threatening.

With this in mind, Garcia et al. developed a heterogeneous class of ligands with the aim to reinforce the antinociceptive activity mediated by μ-opioid receptor (MOR) agonists with an antagonist activity at the σ_1_ receptor that has a well-established role in the treatment of pain. Some pieces of evidence have demonstrated that the antagonist **S1RA** [124,127] is able to potentiate morphine antinociceptive activity without increasing side effects, so that it entered clinical trials for treating different kinds of pain [135,136].

These premises paved the way to the development of a pharmacophore model obtained by overlapping the MOR pharmacophore generated with the “*Auto Pharmacophore generation*” protocol (that enumerates all the pharmacophores compatible with an input molecule considering hydrogen bond acceptors and donors, hydrophobic portions, ionizable groups, and aromatic rings) within the Discovery Studio [137] with the σ_1_ pharmacophoric model developed by Langer [138]. This model was based on the presence of two hydrogen bond acceptors (*HBA1* and *HBA2*), three hydrophobic portions (*HYD1*, *HYD2,* and *HYD34*, obtained by the fusion of two different hydrophobic portions *HBA3* and *HBA4* of Laggner’s pharmacophore), an aromatic ring (*AR*) and two positive ionizable domains (*PI*s).

**Figure 13:**
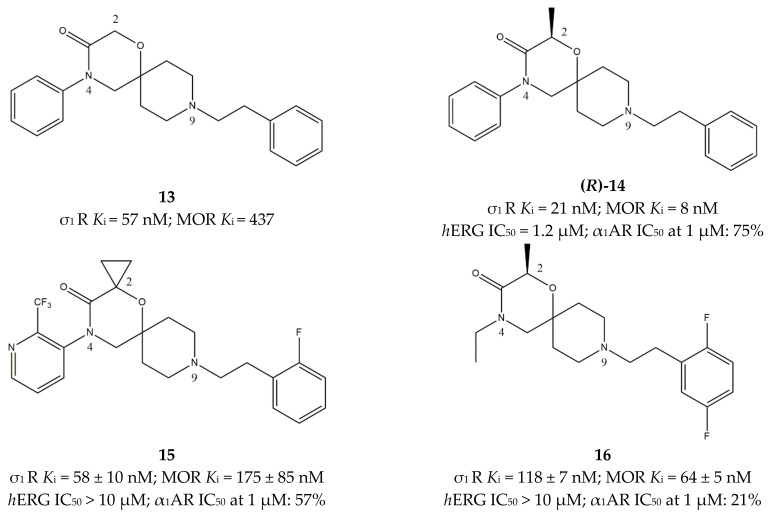
Compounds (**13**–**16**) and their *K*_i_ and IC_50_ values for σ_1_ (σ_1_ R), μ-opioid (MOR) and alpha-1 adrenergic (α_1_AR) receptors and human ether-a-go-go-related gene channel (*h*ERG) [139,140].

Based on this model, several compounds were synthesized, and the 4-aryl-1-oxa-4,9-diazaspiro[5.5]undecane derivative **13** (Figure 13) provided the best results on both targets.

Starting from compound **13**, SAR studies were performed to optimize the structure. In particular, the 2-position on the spiro[5.5]undecane was explored through the introduction of small substituents such as methyl groups (compound **(*R*)-14**, Figure 13), which increased the activity mediated by MOR and σ_1_ receptors, while reducing the activity at the *h*ERG channel, which was responsible for its cardiac toxicity. Moreover, pure enantiomers were tested demonstrating that ***(R*)-14** is 4-fold less active toward *h*ERG and displays a 1-order of magnitude higher affinity for MOR than its (*S*)-counterpart. Elongation of the same alkyl group from methyl to ethyl or isopropyl produced an increase in the activity toward MOR but decreased the activity toward σ_1_ receptors, thus spoiling the beneficial synergy. Substitution with more polar groups produced compounds characterized by a lower affinity than compound **(*R*)-14**, but the activity at the two targets reached a more balanced compromise. Nevertheless, the reduction in lipophilicity did not improve the profile at *h*ERG, which remained quite similar to the inhibition exerted by the more hydrophobic compound **(*R*)-14**.

Another structural exploration was conducted by introducing substituents on the phenyl ring on the 4-position of the 1-oxa-4,9-diazaspiro[5.5]undecane system. In particular, halogens and trifluoromethyl groups in *ortho* positions provided the best results toward the two targets, which was probably because of an increase in steric bulk. On the other hand, no improvement toward *h*ERG inhibition was observed. Position 4 was also explored by introducing heteroaryl groups, and the best results were obtained when 2- or 3-pyridyl systems were inserted. As for the 9-position on the 1-oxa-4,9-diazaspiro[5.5]undecane system, the best results were obtained with the same groups introduced in position 4.

Among all of these compounds, **15** (Figure 13) resulted as the best compromise because of its activity toward the two targets and appreciable selectivity, with the most important achievement represented by the absence of *h*ERG inhibition.

The promising pharmacodynamic profile, together with good solubility, moderate basicity and lipophilicity, and in vitro metabolic stability of compound **15** led the group to study its antinociceptive properties in vivo. Results from the preclinical model, based on the mechanical pressure test on a hindpaw, demonstrated a dose-dependent antinociceptive effect with an ED_50_ of 15 mg/kg after *i.p.* administration. Moreover, local administration of the compound provided promising results [139]. Further optimization of the compound was conducted with the production of new dual ligands with enhanced activity. Replacement of the aromatic ring in the 4-position of the 1-oxa-4,9-diazaspiro[5.5]undecane system with an ethyl group provided encouraging data. In fact, small alkyl groups produced a slightly reduced affinity toward MOR and σ_1_ receptors but considerably improved the profile toward alpha-1 adrenergic receptor (α_1_AR) and *h*ERG, which was likely because of a reduced lipophilicity. As already shown by structural changes leading to compound **14**, different small alkyl substituents were well tolerated at the 2-position (spirocyclopropyl, ethyl, and (*R*)-methyl groups), improving the affinity toward the two targets and the hERG profile. These optimization studies led to compound **16** (Figure 13), which showed good physicochemical properties, high BBB permeability, and no cytotoxic activity in the hepatocarcinoma HepG2 cells. For these reasons, compound **16** was successfully tested in the paw pressure test and in the partial sciatic nerve ligation (PSNL) model in CD1 male mice. Thus, compound **16** was selected as a Phase 1 clinical candidate in the treatment of pain [140].

### 3.7. σ1. Receptor and Cancer in a MTDLs Approach

The σ_1_ receptor is a well-established target in the neurodegenerative field. However, several cancer cell lines (e.g., *lung*: H69, H2019, H510; *breast*: MDA-MB-361, MDA-MB-435, BT20, MCF-7, T47D, MDA-MB-231, MDA-MB-468, SKBR3, BT474; *prostate*: PC3, LNCaP, LAPC4, C4-2, 22Rv1, VCaP, PC3, DU145; *human esophageal squamous cell carcinoma*: KYSE150, KYSE180, EC109; *pancreas*: Panc1; liver: HepG2; *neuroblastoma*: SK-N-BE(2)C) show high levels of σ_1_ proteins or SIGMAR1 transcripts. While the receptor is not an oncogene [141], σ_1_ receptor ligands with claimed antagonist activity exert antiproliferative and growth-inhibiting effects [142].

**Figure 14:**
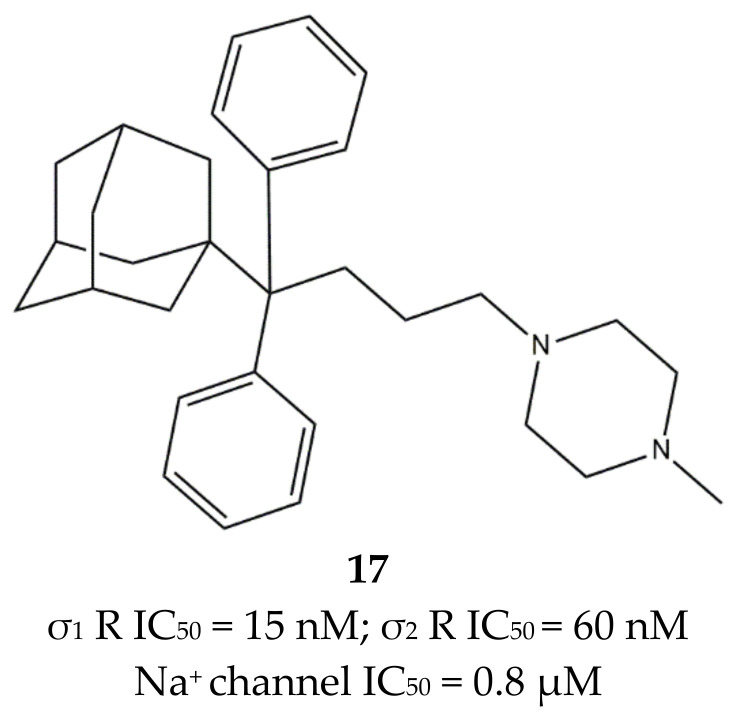
Compound (**17**) structure and its IC_50_ values for σ_1_ receptor (σ_1_ R), σ_2_ receptor (σ_2_ R) and Na^+^ channel [144].

The involvement of the σ_1_ receptor in cancer prompted Riganas et al. to develop (1-adamantyl)diarylalkylamines as σ_1_ receptor ligands whose scaffold was based on two aromatic rings and a hydrophobic adamantane moiety, which were linked to an amine site through alkyl chains of different length. The activity of these ligands was also tested on Na^+^ channels, whose involvement in cancer has been proven [141,143]. Compounds from the butyl series displayed the best profile with good σ_1_ receptor antagonist and weak σ_2_ receptor agonist activities, while all the other compounds showed a lower selectivity for σ_1_ vs. σ_2_. Moreover, all of them showed from micromolar to sub-micromolar affinities for the site 2 of Na^+^ channels. Compound **17** (Figure 14) emerged for its cytotoxic effect in ovarian cancer cells (IGROV-1) and in vitro antiangiogenic activity in normal cell lines such as Human Umbilical Vein Endothelial Cells (HUVEC).

Compound **17** was characterized by high-affinity interaction with the σ_2_ receptor as agonist (i.e., inducer of cytotoxicity) and with the σ_1_ receptor as antagonist, representing the optimal compromise if anticancer agents based on σ receptors-mediated action want to be produced. Additionally, **17** was associated with an analgesic effect against neuropathic pain, which was probably due to the block of Na^+^ channels, thus highlighting the therapeutic potentials of the combination of these actions in one molecule [145].

**Figure 15:**
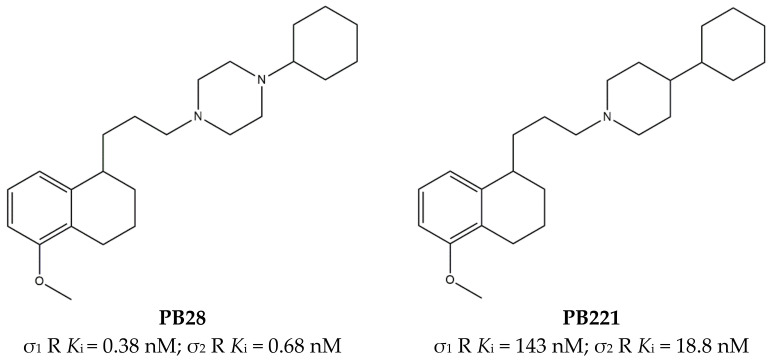
**PB28** and **PB221** structures and *K*_i_ values for σ_1_ receptor (σ_1_ R) and σ_2_ receptor (σ_2_ R) [153,154].

Another ligand conveniently endowed with antagonism at σ_1_ receptors and agonism at σ_2_ receptors is **PB28**, which has been found as a potential anti SARS-CoV-2 agent, and for this reason, researchers have recently reviewed its structure–affinity/activity relationships [144,146]. This compound emerged from structure–affinity relationship (SAfiR) studies that clearly indicated how *N*-alkyl-piperazine connected through a methylene linker to a hydrophobic nucleus (i.e., tetralin), rather than the corresponding *N*-Aryl ones, addressed the σ receptors affinity, and weakened the interaction with dopamine D_2_ and serotonin 5-HT_1A_ receptors [71,147]. With its sub-nanomolar affinity toward both σ receptor subtypes, and the combination of functional activities (σ_1_ antagonism and σ_2_ agonism), **PB28** was tested in several cancer cells (e.g., breast, neuroblastoma, pancreas tumor cell lines) [144]. Moreover, studies performed in pancreatic cancer cell lines (i.e., Panc02, KP02, KCKO, MIA PaCa-2, BxPC-1, and Panc-1) highlighted the increase in ROS production, lysosomal membrane permeabilization (LMP), and mitochondrial superoxide production as **PB28** mechanisms of action that lead to oxidative stress [148]. However, the **PB28** anticancer effect both in vitro and in a preclinical model was not striking despite the promising σ binding profile. With the aim of increasing the cytotoxic activity, structural changes were made and several analogues of **PB28** were synthesized, some of which were endowed with promising anticancer action. As an example, the piperidinyl analogue **PB221** (Figure 15), with reduced σ receptor affinity and a more σ_2_-oriented profile, was thoroughly investigated in CNS tumors and displayed promising results in preclinical models [149]. Additionally, the cytotoxic activity of **PB28** in resistant tumors was increased through the generation of multi-target ligands directed toward σ receptors (the σ_2_ receptor in particular) and P-glycoprotein, with the aim to evade the P-gp efflux whose overexpression is responsible for the drug-resistance phenomenon. While most of the compounds were active both in resistant and wild-type (wt) cancer cells [150], some MTDLs were endowed with the collateral sensitivity phenomenon that is characterized by more potent cytotoxicity in resistant rather than in the wt cells. The promising data obtained prompted to push the MTDL concept further in this context by merging the σ/P-gp scaffold with a metals chelator portion in order to stress the collateral sensitivity property thanks to the more important impact that the chelation of metals, such as iron and copper ions, has on the energy state of cells [151]. Excellent results were obtained with this class of compounds with a thiosemicarbazone structure that showed advantages in preclinical pancreatic tumor models, where the σ-mediated delivery to pancreatic tumors appeared devoid of side effects [152]. However, the σ_1_ involvement was not investigated in the overall effect, as focus on the σ_2_ subtype was given.

**PB28**, also **Haloperidol metabolite II** (Figure 12), is characterized by a σ_1_ receptor antagonism/σ_2_ receptor agonism profile, and it is able to exert modest antiproliferative activity because of the increase in the Intracellular-Free Calcium Levels [Ca^2+^]_i_ and apoptosis induction [155].

**Figure 16:**
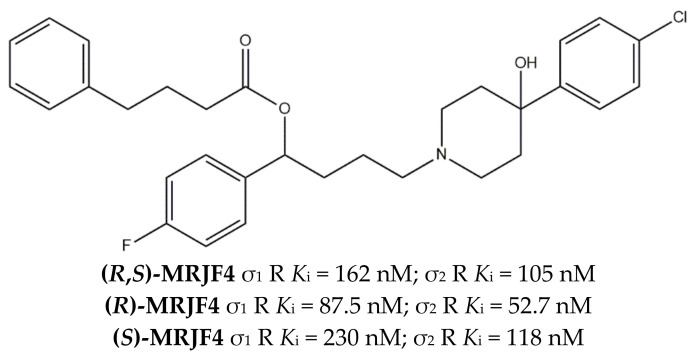
**(R,S)-MRJF4** structure and *K*_i_ values for σ_1_ receptor (σ_1_ R) and σ_2_ receptor (σ_2_ R) [129].

This evidence supported the synthesis of **(*R*,*S*)-MRJF4** (Figure 16), which combined the **Haloperidol metabolite II** profile together with 4-phenylbutyric acid, which inhibits Histone Deacetylase (HDAC) and is involved in the transcription of genes that regulate the cell cycle, with a clear role in cancerogenesis and cancer progression.

The esterification of **Haloperidol metabolite II** with 4-phenylbutyric acid appeared to be detrimental for the affinity toward both classes of receptors, with a drop from nanomolar to low micromolar values. Nevertheless, antiproliferative studies demonstrated that **(*R*,*S*)-MRJF4** is able to produce a more potent antiproliferative effect in LNCaP and PC3 than 4-phenylbutyric acid or **Haloperidol metabolite II** alone or in combination [134].

In the following years, the pure enantiomers **(*R*)-MRJF4** and **(*S*)-MRJF4** were studied, showing that (*R*)-enantiomer has a higher affinity for both σ receptors and higher anticancer activity (Figure 16) [129].

Noteworthy are some studies performed by Nieto et al. about cancer-associated pain in which paclitaxel was used to induce allodynia. This effect was observed in wild-type mice but not in the SIGMAR1 KO mice. Moreover, σ_1_ receptors antagonists, such as **BD1063** and **S1RA** (Table 1), reverted paclitaxel-induced neuropathic pain in wild-type mice [156,157].

Altogether, these studies show that the antiproliferative and growth-inhibiting activity of σ_1_ receptor antagonists can be empowered and likely better oriented when associated to other activities. Importantly, these same antagonists can block cancer-associated pain, which is an effect probably strengthened by the block of Na^+^ channels, thus again supporting the MTDLs approach involving σ_1_ receptors also in the treatment of pain associated to cancer.

## 4. Conclusions and Perspectives

This review aims to point out the state of the art of MTDLs interacting with the σ_1_ receptor. As a result of its pluripotent chaperone activity, the σ_1_ receptor has the potential to interact and modulate several targets involved in a number of diseases and syndromes. The evidence that several compounds already in clinical use (e.g., **Donepezil**, **HP**, etc.) unintentionally bind the σ_1_ receptor has shed light on the already exploited interaction with this protein as a successful strategy in treating illnesses or associated symptoms through a MTDLs approach. **Donepezil**, whose interaction with σ_1_ receptors in vivo has been ascertained by receptor occupancy studies in the human brain [94,158], is co-administered together with Memantine through prescription medicines (i.e., **Namzaric**). On this basis, the rational development of MTDLs with a σ_1_ intentional targeting profile may lead to more important therapeutic actions. The amount of herein listed proteins, which are the object of the MTLDs development in association with the σ_1_ receptor, is not intended to be exhaustive. Other proteins may be taken into consideration in the MTDLs development, considering the wide range of σ_1_ receptor direct and indirect interactors. Cannabinoid type-2 (CB2) receptors are an example, with recent support from computational methods that point out a partial overlap of the σ_1_ and CB2 receptors’ pharmacophores [159]. These data strongly suggest the adoption of a merging approach for the development of dual σ_1_/CB2 receptors ligands for a synergistic exploitation of the pathways activated by the two targets in oncology and neurodegenerative diseases. A number of σ ligands, besides interacting at the two subtypes (see Section 3.7), are able to modulate the efflux pump P-glycoprotein (P-gp) [150,160]. This double action is worthy of being more intentionally addressed, as it may serve as a strategy to face drug-resistant tumors: the compound overcomes P-gp, which is overexpressed in many resistant tumors and exerts its cytotoxic activity (as a σ_1_ antagonist). Importantly, this same double action may also have a role in the treatment of CNS diseases through the bypass of the P-gp at the BBB. The waving interest in σ receptors research increased during the COVD-19 pandemic, when the proteins were found as important key host dependency factors for coronavirus infections. However, while the exploitability of the σ_1_ receptor as a druggable target against coronavirus still needs to be fully investigated and validated, it appears clear that this protein is involved in a plethora of pathways hampered in multifactorial CNS and cancer diseases, rendering the σ_1_ receptor a full-fledged target for the development of multifunctional therapeutics.

## References

[1] Bansal Y., Silakari O. (2014). Multifunctional Compounds: Smart Molecules for Multifactorial Diseases. Eur. J. Med. Chem..

[2] Korcsmáros T., Szalay M.S., Böde C., Kovács I.A., Csermelyt P. (2007). How to Design Multi-Target Drugs: Target Search Options in Cellular Networks. Expert Opin. Drug Discov..

[3] Morphy R., Rankovic Z. (2005). Designed Multiple Ligands. An Emerging Drug Discovery Paradigm. J. Med. Chem..

[4] Walker J.M., Bowen W.D., Walker F.O., Matsumoto R.R., De Costa B., Rice K.C. (1990). Sigma Receptors: Biology and Function. Pharmacol. Rev..

[5] Hellewell S.B., Bowen W.D. (1990). A Sigma-like Binding Site in Rat Pheochromocytoma (PC12) Cells: Decreased Affinity for (+)-Benzomorphans and Lower Molecular Weight Suggest a Different Sigma Receptor Form from That of Guinea Pig Brain. Brain Res..

[6] Alonso G., Phan V., Guillemain I., Saunier M., Legrand A., Anoal M., Maurice T. (2000). Immunocytochemical Localization of the Sigma(1) Receptor in the Adult Rat Central Nervous System. Neuroscience.

[7] Roman F., Pascaud X., Chomette G., Bueno L., Junien J.L. (1989). Autoradiographic Localization of Sigma Opioid Receptors in the Gastrointestinal Tract of the Guinea Pig. Gastroenterology.

[8] Su T.P., Wu X.Z. (1990). Guinea Pig Vas Deferens Contains ρ but Not Phencyclidine Receptors. Neurosci. Lett..

[9] Hellewell S.B., Bruce A., Feinstein G., Orringer J., Williams W., Bowen W.D. (1994). Rat Liver and Kidney Contain High Densities of Sigma 1 and Sigma 2 Receptors: Characterization by Ligand Binding and Photoaffinity Labeling. Eur. J. Pharmacol..

[10] Ela C., Barg J., Vogel Z., Hasin Y., Eilam Y. (1994). Sigma Receptor Ligands Modulate Contractility, Ca++ Influx and Beating Rate in Cultured Cardiac Myocytes. J. Pharmacol. Exp. Ther..

[11] Wolfe S.A.J., Culp S.G., De Souza E.B. (1989). Sigma-Receptors in Endocrine Organs: Identification, Characterization, and Autoradiographic Localization in Rat Pituitary, Adrenal, Testis, and Ovary. Endocrinology.

[12] Hayashi T., Su T.P. (2007). Sigma-1 Receptor Chaperones at the ER- Mitochondrion Interface Regulate Ca2+ Signaling and Cell Survival. Cell.

[13] Gasparre G., Abate C., Carlucci R., Berardi F., Cassano G. (2017). The σ1 Receptor Agonist (+)-Pentazocine Increases Store-Operated Ca2+ Entry in MCF7σ1 and SK-N-SH Cell Lines. Pharmacol. Rep..

[14] Mori T., Hayashi T., Hayashi E., Su T.P. (2013). Sigma-1 Receptor Chaperone at the ER-Mitochondrion Interface Mediates the Mitochondrion-ER-Nucleus Signaling for Cellular Survival. PLoS ONE.

[15] Weng T.-Y., Tsai S.-Y.A., Su T.-P. (2017). Roles of Sigma-1 Receptors on Mitochondrial Functions Relevant to Neurodegenerative Diseases. J. Biomed. Sci..

[16] Su T.P., Su T.C., Nakamura Y., Tsai S.Y. (2016). The Sigma-1 Receptor as a Pluripotent Modulator in Living Systems. Trends Pharmacol. Sci..

[17] Schmidt H.R., Kruse A.C. (2019). The Molecular Function of σ Receptors: Past, Present, and Future. Trends Pharmacol. Sci..

[18] Vela J.M. (2020). Repurposing Sigma-1 Receptor Ligands for COVID-19 Therapy?. Front. Pharmacol..

[19] Gordon D.E., Gordon D.E., Hiatt J., Bouhaddou M., Rezelj V.V., Ulferts S. (2020). Comparative Host-Coronavirus Protein Interaction Networks Reveal Pan-Viral Disease Mechanisms. Science.

[20] Mishina M., Ohyama M., Ishii K., Kitamura S., Kimura Y., Oda K.I., Kawamura K., Sasaki T., Kobayashi S., Katayama Y. (2008). Low Density of Sigma1 Receptors in Early Alzheimer’s Disease. Ann. Nucl. Med..

[21] Huang Y., Zheng L., Halliday G., Dobson-Stone C., Wang Y., Tang H.-D., Cao L., Deng Y.-L., Wang G., Zhang Y.-M. (2011). Genetic Polymorphisms in Sigma-1 Receptor and Apolipoprotein E Interact to Influence the Severity of Alzheimers Disease. Curr. Alzheimer Res..

[22] Fehér Á., Juhász A., László A., Kálmán J., Pákáski M., Kálmán J., Janka Z. (2012). Association between a Variant of the Sigma-1 Receptor Gene and Alzheimer’s Disease. Neurosci. Lett..

[23] Christ M.G., Clement A.M., Behl C. (2020). The Sigma-1 Receptor at the Crossroad of Proteostasis, Neurodegeneration, and Autophagy. Trends Neurosci..

[24] Hanner M., Moebius F.F., Flandorfer A., Knaus H.G., Striessnig J., Kempner E., Glossmann H. (1996). Purification, Molecular Cloning, and Expression of the Mammalian Sigma1-Binding Site. Proc. Natl. Acad. Sci. USA.

[25] Schmidt H.R., Zheng S., Gurpinar E., Koehl A., Manglik A., Kruse A.C. (2016). Crystal Structure of the Human σ1 Receptor. Nature.

[26] Schmidt H.R., Betz R.M., Dror R.O., Kruse A.C. (2018). Structural Basis for σ(1) Receptor Ligand Recognition. Nat. Struct. Mol. Biol..

[27] Glennon R. (2005). Pharmacophore Identification for Sigma-1 (σ1) Receptor Binding: Application of the “Deconstruction-Reconstruction-Elaboration” Approach. Mini-Rev. Med. Chem..

[28] Niso M., Mosier P.D., Marottoli R., Ferorelli S., Cassano G., Gasparre G., Leopoldo M., Berardi F., Abate C. (2019). High-Affinity Sigma-1 (σ1) Receptor Ligands Based on the σ 1 Antagonist PB212. Future Med Chem..

[29] Mishra A.K., Mavlyutov T., Singh D.R., Biener G., Yang J., Oliver J.A., Ruoho A., Raicu V. (2015). The Sigma-1 Receptors Are Present in Monomeric and Oligomeric Forms in Living Cells in the Presence and Absence of Ligands. Biochem. J..

[30] Albayrak Y., Hashimoto K. (2017). Sigma-1 Receptor Agonists and Their Clinical Implications in Neuropsychiatric Disorders. Adv. Exp. Med. Biol..

[31] Ferris R.M., Tang FL M., Chang K.J., Russell A. (2019). Evidence That the Potential Antipsychotic Agent Rimcazole (BW 234U) Is a Specific, Competitive Antagonist of Sigma Sites in Brain. J. Chem. Inf. Model..

[32] Gilmore D.L., Liu Y., Matsumoto R.R. (2004). Review of the Pharmacological and Clinical Profile of Rimcazole. CNS Drug Rev..

[33] Schoenwald R.D., Barfknecht C.F., Shirolkar S., Xia E. (1995). The Effects of Sigma Ligands on Protein Release from Lacrimal Acinar Cells: A Potential Agonist/Antagonist Assay. Life Sci..

[34] Taylor D.P., Eison M.S., Moon S.L., Schlemmer R.F.J., Shukla U.A., VanderMaelen C.P., Yocca F.D., Gallant D.J., Behling S.H., Boissard C.G. (1993). A Role for Sigma Binding in the Antipsychotic Profile of BMY 14802?. NIDA Res. Monogr..

[35] Yano H., Bonifazi A., Xu M., Guthrie D.A., Schneck S.N., Abramyan A.M., Fant A.D., Hong W.C., Newman A.H., Shi L. (2018). Pharmacological Profiling of Sigma 1 Receptor Ligands by Novel Receptor Homomer Assays. Neuropharmacology.

[36] Gómez-Soler M., Fernández-Dueñas V., Portillo-Salido E., Pérez P., Zamanillo D., Vela J.M., Burgueño J., Ciruela F. (2014). Predicting the Antinociceptive Efficacy of σ1 Receptor Ligands by a Novel Receptor Fluorescence Resonance Energy Transfer (FRET) Based Biosensor. J. Med. Chem..

[37] Fontanilla D., Johannessen M., Hajipour A.R., Cozzi N.V., Jackson M.B., Ruoho A.E. (2009). The Hallucinogen N,N-Dimethyltryptamine (DMT) Is an Endogenous Sigma-1 Receptor Regulator. Science.

[38] Johannessen M., Fontanilla D., Mavlyutov T., Ruoho A.E., Jackson M.B. (2011). Antagonist Action of Progesterone at σ-Receptors in the Modulation of Voltage-Gated Sodium Channels. Am. J. Physiol. Cell Physiol..

[39] Su T.P., London E.D., Jaffe J.H. (1988). Steroid Binding at σ Receptors Suggests a Link between Endocrine, Nervous, and Immune Systems. Science.

[40] Su T.P. (1982). Evidence for Sigma Opioid Receptor: Binding of [3H]SKF-10047 to Etorphine-Inaccessible Sites in Guinea-Pig Brain. J. Pharmacol. Exp. Ther..

[41] Matsumoto R.R., Nguyen L., Kaushal N., Robson M.J. (2014). Sigma (σ) Receptors as Potential Therapeutic Targets to Mitigate Psychostimulant Effects. Adv. Pharmacol..

[42] Ishiguro H., Ohtsuki T., Toru M., Itokawa M., Aoki J., Shibuya H., Kurumaji A., Okubo Y., Iwawaki A., Ota K. (1998). Association between Polymorphisms in the Type 1 Sigma Receptor Gene and Schizophrenia. Neurosci. Lett..

[43] Uchida N., Ujike H., Nakata K., Takaki M., Nomura A., Katsu T., Tanaka Y., Imamura T., Sakai A., Kuroda S. (2003). No Association between the Sigma Receptor Type 1 Gene and Schizophrenia: Results of Analysis and Meta-Analysis of Case-Control Studies. BMC Psychiatry.

[44] Narita N., Hashimoto K., Tomitaka S.I., Minabe Y. (1996). Interactions of Selective Serotonin Reuptake Inhibitors with Subtypes of σ Receptors in Rat Brain. Eur. J. Pharmacol..

[45] Urani A., Roman F.J., Phan V.L., Su T.P., Maurice T. (2001). The Antidepressant-like Effect Induced by Sigma(1)-Receptor Agonists and Neuroactive Steroids in Mice Submitted to the Forced Swimming Test. J. Pharmacol. Exp. Ther..

[46] Skuza G., Szymańska M., Budziszewska B., Abate C., Berardi F. (2011). Effects of PB190 and PB212, New σ Receptor Ligands, on Glucocorticoid Receptor-Mediated Gene Transcription in LMCAT Cells. Pharmacol. Rep..

[47] Maurice T., Roman F.J., Privat A. (1996). Modulation by neurosteroids of the in vivo (+)-[3H]SKF-10,047 binding to σ1 receptors in the mouse forebrain. J. Neurosci. Res..

[48] Matsuno K., Nakazawa M., Okamoto K., Kawashima Y., Mita S. (1996). Binding Properties of SA4503, a Novel and Selective s Receptor Agonist. Eur. J. Pharmacol..

[49] Senda T., Matsuno K., Okamoto K., Kobayashi T., Nakata K., Mita S. (1996). Ameliorating Effect of SA4503, a Novel σ1 Receptor Agonist, on Memory Impairments Induced by Cholinergic Dysfunction in Rats. Eur. J. Pharmacol..

[50] Skuza G., Rogóz Z. (2002). A Potential Antidepressant Activity of SA4503, a Selective σ1 Receptor Agonist. Behav. Pharmacol..

[51] Hirano K., Tagashira H., Fukunaga K. (2014). Cardioprotective Effect of the Selective Sigma-1 Receptor Agonist, SA4503. Yakugaku Zasshi.

[52] Yamashita D., Sun G.-W., Cui Y., Mita S., Otsuki N., Kanzaki S., Nibu K.I., Ogawa K., Matsunaga T. (2015). Neuroprotective Effects of Cutamesine, a Ligand of the Sigma-1 Receptor Chaperone, against Noise-Induced Hearing Loss. J. Neurosci. Res..

[53] Berardi F., Ferorelli S., Abate C., Pedone M.P., Colabufo N.A., Contino M., Perrone R. (2005). Methyl Substitution on the Piperidine Ring of N-[ω-(6- Methoxynaphthalen-1-Yl)Alkyl] Derivatives as a Probe for Selective Binding and Activity at the σ1 Receptor. J. Med. Chem..

[54] Moritz C., Berardi F., Abate C., Peri F. (2015). Live Imaging Reveals a New Role for the Sigma-1 (σ1) Receptor in Allowing Microglia to Leave Brain Injuries. Neurosci. Lett..

[55] Skuza G., Sadaj W., Kabziński M., Cassano G., Gasparre G., Abate C., Berardi F. (2014). The Effects of New Sigma (σ) Receptor Ligands, PB190 and PB212, in the Models Predictive of Antidepressant Activity. Pharmacol. Rep..

[56] Su T.P., Wu X.Z., Cone E.J., Shukla K., Gund T.M., Dodge A.L., Parish D.W. (1991). Sigma Compounds Derived from Phencyclidine: Identification of PRE-084, a New, Selective Sigma Ligand. J. Pharmacol. Exp. Ther..

[57] Maurice T., Su T.P., Parish D.W., Nabeshima T., Privat A. (1994). PRE-084, a σ Selective PCP Derivative, Attenuates MK-801-Induced Impairment of Learning in Mice. Pharmacol. Biochem. Behav..

[58] Maurice T. (2001). Beneficial Effect of the σ1 Receptor Agonist PRE-084 against the Spatial Learning Deficits in Aged Rats. Eur. J. Pharmacol..

[59] Okuyama S., Nakazato A. (1996). NE-100: A Novel Sigma Receptor Antagonist. CNS Drug Rev..

[60] Chaki S., Tanaka M., Muramatsu M., Otomo S. (1994). NE-100, a Novel Potent σ Ligand, Preferentially Binds to Σ1 Binding Sites in Guinea Pig Brain. Eur. J. Pharmacol..

[61] Spinelli F., Haider A., Toscano A., Pati M.L., Keller C., Berardi F., Colabufo N.A., Abate C., Ametamey S.M. (2018). Synthesis, Radiolabelling, and Evaluation of [(11)C]PB212 as a Radioligand for Imaging Sigma-1 Receptors Using PET. Am. J. Nucl. Med. Mol. Imaging.

[62] Maeda D.Y., Williams W., Bowen W.D., Coop A. (2000). A Sigma-1 Receptor Selective Analogue of BD1008. A Potential Substitute for (+)-Opioids in Sigma Receptor Binding Assays. Bioorganic Med. Chem. Lett..

[63] Sahlholm K., Århem P., Fuxe K., Marcellino D. (2013). The Dopamine Stabilizers ACR16 and ()-OSU6162 Display Nanomolar Affinities at the σ-1 Receptor. Mol. Psychiatry.

[64] James M.L., Shen B., Zavaleta C.L., Nielsen C.H., Mesangeau C., Vuppala P.K., Chan C., Avery B.A., Fishback J.A., Matsumoto R.R. (2012). New Positron Emission Tomography (PET) Radioligand for Imaging σ-1 Receptors in Living Subjects. J. Med. Chem..

[65] James M.L., Shen B., Nielsen C.H., Behera D., Buckmaster C.L., Mesangeau C., Zavaleta C., Vuppala P.K., Jamalapuram S., Avery B.A. (2014). Evaluation of σ-1 Receptor Radioligand 18F-FTC-146 in Rats and Squirrel Monkeys Using PET. J. Nucl. Med..

[66] Shen B., Park J.H., Hjørnevik T., Cipriano P.W., Yoon D., Gulaka P.K., Holly D., Behera D., Avery B.A., Gambhir S.S. (2017). Radiosynthesis and First-In-Human PET/MRI Evaluation with Clinical-Grade [18F]FTC-146. Mol. Imaging Biol..

[67] Díaz J.L., Zamanillo D., Corbera J., Baeyens J.M., Maldonado R., Pericàs M.A., Vela J.M., Torrens A. (2009). Selective Sigma-1 (Sigma1) Receptor Antagonists: Emerging Target for the Treatment of Neuropathic Pain. Cent. Nerv. Syst. Agents Med. Chem..

[68] Abadias M., Escriche M., Vaqué A., Sust M., Encina G. (2013). Safety, Tolerability and Pharmacokinetics of Single and Multiple Doses of a Novel Sigma-1 Receptor Antagonist in Three Randomized Phase I Studies. Br. J. Clin. Pharmacol..

[69] Matsumoto R.R., Bowen W.D., Tom M.A., Vo V.N., Truong D.D., De Costa B.R. (1995). Characterization of Two Novel σ Receptor Ligands: Antidystonic Effects in Rats Suggest σ Receptor Antagonism. Eur. J. Pharmacol..

[70] Matsumoto R.R., McCracken K.A., Friedman M.J., Pouw B., De Costa B.R., Bowen W.D. (2001). Conformationally Restricted Analogs of BD1008 and an Antisense Oligodeoxynucleotide Targeting σ1 Receptors Produce Anti-Cocaine Effects in Mice. Eur. J. Pharmacol..

[71] Perrone R., Berardi F., Colabufo N.A., Leopoldo M., Abate C., Tortorella V. (2000). N-Aryl or N-Alkylpiperazine Derivatives: The Role of N-Substituent on σ1, σ2, 5-HT1A and D2 Receptor Affinity. Med. Chem. Res..

[72] Lever J.R., Gustafson J.L., Xu R., Allmon R.L., Lever S.Z. (2006). σ1 and σ2 Receptor Binding Affinity and Selectivity of SA4503 and Fluoroethyl SA4503. Synapse.

[73] Garces-Ramirez L., Green J.L., Hiranita T., Kopajtic T.A., Mereu M., Thomas A.M., Mesangeau C., Narayanan S., McCurdy C.R., Katz J.L. (2011). Sigma Receptor Agonists: Receptor Binding and Effects on Mesolimbic Dopamine Neurotransmission Assessed by Microdialysis. Biol. Psychiatry.

[74] Colabufo N.A., Berardi F., Contino M., Perrone R., Tortorella V. (2003). A New Method for Evaluating σ2 Ligand Activity in the Isolated Guinea-Pig Bladder. Naunyn. Schmiedebergs. Arch. Pharmacol..

[75] Johnston T.H., Geva M., Steiner L., Orbach A., Papapetropoulos S., Savola J.M., Reynolds I.J., Ravenscroft P., Hill M., Fox S.H. (2019). Pridopidine, a Clinic-Ready Compound, Reduces 3,4-Dihydroxyphenylalanine-Induced Dyskinesia in Parkinsonian Macaques. Mov. Disord..

[76] Romero L., Zamanillo D., Nadal X., Sánchez-Arroyos R., Rivera-Arconada I., Dordal A., Montero A., Muro A., Bura A., Segalés C. (2012). Pharmacological Properties of S1RA, a New Sigma-1 Receptor Antagonist That Inhibits Neuropathic Pain and Activity-Induced Spinal Sensitization. Br. J. Pharmacol..

[77] Lecanu L., Tillement L., McCourty A., Rammouz G., Yao W., Greeson J., Papadopoulos V. (2012). Dimethyl-Carbamic Acid 2,3-Bis-Dimethylcarbamoyloxy-6-(4-Ethyl-Piperazine- 1-Carbonyl)-Phenyl Ester: A Novel Multi-Target Therapeutic Approach to Neuroprotection. Med. Chem..

[78] Caccamo A., Oddo S., Billings L.M., Green K.N., Martinez-Coria H., Fisher A., LaFerla F.M. (2006). M1 Receptors Play a Central Role in Modulating AD-like Pathology in Transgenic Mice. Neuron.

[79] Medeiros R., Kitazawa M., Caccamo A., Baglietto-Vargas D., Estrada-Hernandez T., Cribbs D.H., Fisher A., Laferla F.M. (2011). Loss of Muscarinic M1 Receptor Exacerbates Alzheimer’s Disease-like Pathology and Cognitive Decline. Am. J. Pathol..

[80] Vamvakidès A. (2002). [Anticonvulsant and forced swim anti-immobility effects of tetrahydro-N, N-dimethyl-2,2-diphenyl-3-furanemethanamine (AE37): Common action mechanism?]. Ann. Pharm. Fr..

[81] Vamvakidès A. (2002). [Mechanism of action of tetrahydro-N, N-dimethyl-5, 5-diphenyl-3-furanemethanamine, a putative nootropic, anti-epileptic and antidepressant compound]. Ann. Pharm. Fr..

[82] Fisher A., Bezprozvanny I., Wu L., Ryskamp D.A., Bar-Ner N., Natan N., Brandeis R., Elkon H., Nahum V., Gershonov E. (2016). AF710B, a Novel M1/σ1 Agonist with Therapeutic Efficacy in Animal Models of Alzheimer’s Disease. Neurodegener. Dis..

[83] Chow H.M., Guo D., Zhou J.C., Zhang G.Y., Li H.F., Herrup K., Zhang J. (2014). CDK5 Activator Protein P25 Preferentially Binds and Activates GSK3β. Proc. Natl. Acad. Sci. USA.

[84] Lahmy V., Meunier J., Malmström S., Naert G., Givalois L., Kim S.H., Villard V., Vamvakides A., Maurice T. (2013). Blockade of Tau Hyperphosphorylation and Aβ 1-42 Generation by the Aminotetrahydrofuran Derivative ANAVEX2-73, a Mixed Muscarinic and σ 1 Receptor Agonist, in a Nontransgenic Mouse Model of Alzheimer’s Disease. Neuropsychopharmacology.

[85] Espallergues J., Lapalud P., Christopoulos A., Avlani V.A., Sexton P.M., Vamvakides A., Maurice T. (2007). Involvement of the Sigma1 (σ1) Receptor in the Anti-Amnesic, but Not Antidepressant-like, Effects of the Aminotetrahydrofuran Derivative ANAVEX1-41. Br. J. Pharmacol..

[86] Behensky A.A., Yasny I.E., Shuster A.M., Seredenin S.B., Petrov A.V., Cuevas J. (2013). Stimulation of Sigma Receptors with Afobazole Blocks Activation of Microglia and Reduces Toxicity Caused by Amyloid-Β25-35. J. Pharmacol. Exp. Ther..

[87] Behensky A.A., Katnik C., Yin H., Cuevas J. (2019). Activation of Sigma Receptors with Afobazole Modulates Microglial, but Not Neuronal, Apoptotic Gene Expression in Response to Long-Term Ischemia Exposure. Front. Neurosci..

[88] Hall H., Iulita M.F., Gubert P., Flores Aguilar L., Ducatenzeiler A., Fisher A., Cuello A.C. (2018). AF710B, an M1/Sigma-1 Receptor Agonist with Long-Lasting Disease-Modifying Properties in a Transgenic Rat Model of Alzheimer’s Disease. Alzheimer’s Dement..

[89] Rochais C., Lecoutey C., Gaven F., Giannoni P., Hamidouche K., Hedou D., Dubost E., Genest D., Yahiaoui S., Freret T. (2015). Novel Multitarget-Directed Ligands (MTDLs) with Acetylcholinesterase (AChE) Inhibitory and Serotonergic Subtype 4 Receptor (5-HT4R) Agonist Activities As Potential Agents against Alzheimer’s Disease: The Design of Donecopride. J. Med. Chem..

[90] Lalut J., Santoni G., Karila D., Lecoutey C., Davis A., Nachon F., Silman I., Sussman J., Weik M., Maurice T. (2019). Novel Multitarget-Directed Ligands Targeting Acetylcholinesterase and σ1 Receptors as Lead Compounds for Treatment of Alzheimer’s Disease: Synthesis, Evaluation, and Structural Characterization of Their Complexes with Acetylcholinesterase. Eur. J. Med. Chem..

[91] Kato K., Hayako H., Ishihara Y., Marui S., Iwane M., Miyamoto M. (1999). TAK-147, an Acetylcholinesterase Inhibitor, Increases Choline Acetyltransferase Activity in Cultured Rat Septal Cholinergic Neurons. Neurosci. Lett..

[92] Maurice T., Meunier J., Feng B., Ieni J., Monaghan D.T. (2006). Interaction with σ1 Protein, but Not N-Methyl-D-Aspartate Receptor, Is Involved in the Pharmacological Activity of Donepezil. J. Pharmacol. Exp. Ther..

[93] Meunier J., Ieni J., Maurice T. (2006). The Anti-Amnesic and Neuroprotective Effects of Donepezil against Amyloid Β 25-35 Peptide-Induced Toxicity in Mice Involve an Interaction with the σ1 Receptor. Br. J. Pharmacol..

[94] Ramakrishnan N.K., Visser A.K.D., Schepers M., Luurtsema G., Nyakas C.J., Elsinga P.H., Ishiwata K., Dierckx R.A.J.O., Van Waarde A. (2014). Dose-Dependent Sigma-1 Receptor Occupancy by Donepezil in Rat Brain Can Be Assessed with 11C-SA4503 and MicroPET. Psychopharmacology.

[95] Maurice T. (2016). Protection by Sigma-1 Receptor Agonists Is Synergic with Donepezil, but Not with Memantine, in a Mouse Model of Amyloid-Induced Memory Impairments. Behav. Brain Res..

[96] Ganapathy M.E., Prasad P.D., Huang W., Seth P., Leibach F.H., Ganapathy V. (1999). Molecular and Ligand-Binding Characterization of the Sigma-Receptor in the Jurkat Human T Lymphocyte Cell Line. J. Pharmacol. Exp. Ther..

[97] Castañeda-Arriaga R., Alvarez-Idaboy J.R. (2014). Lipoic Acid and Dihydrolipoic Acid. A Comprehensive Theoretical Study of Their Antioxidant Activity Supported by Available Experimental Kinetic Data. J. Chem. Inf. Model..

[98] Fava A., Pirritano D., Plastino M., Cristiano D., Puccio G., Colica C., Ermio C., De Bartolo M., Mauro G., Bosco D. (2013). The Effect of Lipoic Acid Therapy on Cognitive Functioning in Patients with Alzheimer’s Disease. J. Neurodegener. Dis..

[99] Estrada M., Pérez C., Soriano E., Laurini E., Romano M., Pricl S., Morales-García J.A., Pérez-Castillo A., Rodríguez-Franco M.I. (2016). New Neurogenic Lipoic-Based Hybrids as Innovative Alzheimer’s Drugs with σ-1 Agonism and β-Secretase Inhibition. Future Med. Chem..

[100] Estrada Valencia M., Herrera-Arozamena C., de Andrés L., Pérez C., Morales-García J.A., Pérez-Castillo A., Ramos E., Romero A., Viña D., Yáñez M. (2018). Neurogenic and Neuroprotective Donepezil-Flavonoid Hybrids with Sigma-1 Affinity and Inhibition of Key Enzymes in Alzheimer’s Disease. Eur. J. Med. Chem..

[101] Estrada-Valencia M., Herrera-Arozamena C., Pérez C., Viña D., Morales-García J.A., Pérez-Castillo A., Ramos E., Romero A., Laurini E., Pricl S. (2019). New Flavonoid–N,N-Dibenzyl(N-Methyl)Amine Hybrids: Multi-Target-Directed Agents for Alzheimer’s Disease Endowed with Neurogenic Properties. J. Enzym. Inhib. Med. Chem..

[102] Quirion R., Chicheportiche R., Contreras P.C., Johnson K.M., Lodge D., William Tam S., Woods J.H., Zukin S.R. (1987). Classification and Nomenclature of Phencyclidine and Sigma Receptor Sites. Trends Neurosci..

[103] Quirion R., Bowen W.D., Itzhak Y., Junien J.L., Musacchio J.M., Rothman R.B., Su T.P., Tam S.W., Taylor D.P. (1992). A Proposal for the Classification of Sigma Binding Sites. Trends Pharmacol. Sci..

[104] Pabba M., Sibille E. (2015). Sigma-1 and N-Methyl-D-Aspartate Receptors: A Partnership with Beneficial Outcomes. Mol. Neuropsychiatry.

[105] Bergeron R., de Montigny C., Debonnel G. (1995). Biphasic Effects of Sigma Ligands on the Neuronal Response to N-Methyl-D-Aspartate. Naunyn. Schmiedebergs. Arch. Pharmacol..

[106] Liang X., Wang R.Y. (1998). Biphasic Modulatory Action of the Selective Sigma Receptor Ligand SR 31742A on N-Methyl-D-Aspartate-Induced Neuronal Responses in the Frontal Cortex. Brain Res..

[107] Fletcher E.J., Church J., Abdel-Hamid K., MacDonald J.F. (1995). Blockade by Sigma Site Ligands of N-methyl-D-aspartate-evoked Responses in Rat and Mouse Cultured Hippocampal Pyramidal Neurones. Br. J. Pharmacol..

[108] Martina M., Turcotte M.E.B., Halman S., Bergeron R. (2007). The Sigma-1 Receptor Modulates NMDA Receptor Synaptic Transmission and Plasticity via SK Channels in Rat Hippocampus. J. Physiol..

[109] Van der Schyf C.J., Squier G.J., Coetzee W.A. (1986). Characterization of NGP 1-01, an Aromatic Polycyclic Amine, as a Calcium Antagonist. Pharmacol. Res. Commun..

[110] Geldenhuys J.W., Van der Schyf C.J. (2013). Rationally Designed Multi-Targeted Agents Against Neurodegenerative Diseases. Curr. Med. Chem..

[111] Kassiou M., Nguyen V.H., Knott R., Christie M.J., Hambley T.W. (1996). Trishomocubanes, a New Class of Selective and High Affinity Ligands for the Sigma Binding Site. Bioorganic Med. Chem. Lett..

[112] Nguyen V.H., Kassiou M., Johnston G.A.R., Christie M.J. (1996). Comparison of Binding Parameters of σ1 and σ2 Binding Sites in Rat and Guinea Pig Brain Membranes: Novel Subtype-Selective Trishomocubanes. Eur. J. Pharmacol..

[113] Geldenhuys W.J., Malan S.F., Bloomquist J.R., Marchand A.P., Van Der Schyf C.J. (2005). Pharmacology and Structure-Activity Relationships of Bioactive Polycyclic Cage Compounds: A Focus on Pentacycloundecane Derivatives. Med. Res. Rev..

[114] Kiewert C., Hartmann J., Stoll J., Thekkumkara T.J., Van Der Schyf C.J., Klein J. (2006). NGP1-01 Is a Brain-Permeable Dual Blocker of Neuronal Voltage- and Ligand-Operated Calcium Channels. Neurochem. Res..

[115] Kornhuber J., Schoppmeyer K., Riederer P. (1993). Affinity of 1-Aminoadamantanes for the σ Binding Site in Post-Mortem Human Frontal Cortex. Neurosci. Lett..

[116] Kornhuber J., Bormann J., Hübers M., Rusche K., Riederer P. (1991). Effects of the 1-Amino-Adamantanes at the MK-801-Binding Site of the NMDA-Receptor-Gated Ion Channel: A Human Postmortem Brain Study. Eur. J. Pharmacol. Mol. Pharmacol..

[117] Peeters M., Romieu P., Maurice T., Su T.P., Maloteaux J.M., Hermans E. (2004). Involvement of the Sigma1 Receptor in the Modulation of Dopaminergic Transmission by Amantadine. Eur. J. Neurosci..

[118] Moreno E., Moreno-Delgado D., Navarro G., Hoffmann H.M., Fuentes S., Rosell-Vilar S., Gasperini P., Rodríguez-Ruiz M., Medrano M., Mallol J. (2014). Cocaine Disrupts Histamine H3 Receptor Modulation of Dopamine D1 Receptor Signaling: σ1-D1-H3 Receptor Complexes as Key Targets for Reducing Cocaine’s Effects. J. Neurosci..

[119] Navarro G., Moreno E., Bonaventura J., Brugarolas M., Farré D., Aguinaga D., Mallol J., Cortés A., Casadó V., Lluís C. (2013). Cocaine Inhibits Dopamine D2 Receptor Signaling via Sigma-1-D2 Receptor Heteromers. PLoS ONE.

[120] Sambo D.O., Lin M., Owens A., Lebowitz J.J., Richardson B., Jagnarine D.A., Shetty M., Rodriquez M., Alonge T., Ali M. (2017). The Sigma-1 Receptor Modulates Methamphetamine Dysregulation of Dopamine Neurotransmission. Nat. Commun..

[121] Sahlholm K., Sijbesma J.W.A., Maas B., Kwizera C., Marcellino D., Ramakrishnan N.K., Dierckx R.A.J.O., Elsinga P.H., Van Waarde A. (2015). Pridopidine Selectively Occupies Sigma-1 Rather than Dopamine D2 Receptors at Behaviorally Active Doses. Psychopharmacology.

[122] Francardo V., Geva M., Bez F., Denis Q., Steiner L., Hayden M.R., Cenci M.A. (2019). Pridopidine Induces Functional Neurorestoration Via the Sigma-1 Receptor in a Mouse Model of Parkinson’s Disease. Neurotherapeutics.

[123] Ryskamp D., Wu L., Wu J., Kim D., Rammes G., Geva M., Hayden M., Bezprozvanny I. (2019). Pridopidine Stabilizes Mushroom Spines in Mouse Models of Alzheimer’s Disease by Acting on the Sigma-1 Receptor. Neurobiol. Dis..

[124] Kadnikov I.A., Voronin M.V., Seredenin S.B. (2015). Cytoprotective Effect of Afobazole and Its Main Metabolite M-11. Bull. Exp. Biol. Med..

[125] Voronin M.V., Kadnikov I.A. (2016). Contribution of Sigma-1 Receptor to Cytoprotective Effect of Afobazole. Pharmacol. Res. Perspect..

[126] Voronin M.V., Kadnikov I.A., Voronkov D.N., Seredenin S.B. (2019). Chaperone Sigma1R Mediates the Neuroprotective Action of Afobazole in the 6-OHDA Model of Parkinson’s Disease. Sci. Rep..

[127] Matsumoto R.R., Pouw B. (2000). Correlation between Neuroleptic Binding to σ1 and σ2 Receptors and Acute Dystonic Reactions. Eur. J. Pharmacol..

[128] Cobos E.J., Del Pozo E., Baeyens J.M. (2007). Irreversible Blockade of Sigma-1 Receptors by Haloperidol and Its Metabolites in Guinea Pig Brain and SH-SY5Y Human Neuroblastoma Cells. J. Neurochem..

[129] Sozio P., Fiorito J., Di Giacomo V., Di Stefano A., Marinelli L., Cacciatore I., Cataldi A., Pacella S., Turkez H., Parenti C. (2015). Haloperidol Metabolite II Prodrug: Asymmetric Synthesis and Biological Evaluation on Rat C6 Glioma Cells. Eur. J. Med. Chem..

[130] Korpi E.R., Costakos D.T., Wyatt R.J. (1985). Interconversions of Haloperidol and Reduced Haloperidol in Guinea Pig and Rat Liver Microsomes. Biochem. Pharmacol..

[131] Usuki E., Van Der Schyf C.J., Castagnoli N. (1998). Metabolism of Haloperidol and Its Tetrahydrophyridine Dehydration Product HPTP. Drug Metab. Rev..

[132] Maurice T., Martin-Fardon R., Romieu P., Matsumoto R.R. (2002). Sigma1 (σ1) Receptor Antagonists Represent a New Strategy against Cocaine Addiction and Toxicity. Neurosci. Biobehav. Rev..

[133] Guitart X., Codony X., Monroy X. (2004). Sigma Receptors: Biology and Therapeutic Potential. Psychopharmacology.

[134] Marrazzo A., Fiorito J., Zappal L., Prezzavento O., Ronsisvalle S., Pasquinucci L., Scoto G.M., Bernardini R., Ronsisvalle G. (2011). Antiproliferative Activity of Phenylbutyrate Ester of Haloperidol Metabolite II [(±)-MRJF4] in Prostate Cancer Cells. Eur. J. Med. Chem..

[135] Vidal-Torres A., De La Puente B., Rocasalbas M., Touriño C., Andreea Bura S., Fernández-Pastor B., Romero L., Codony X., Zamanillo D., Buschmann H. (2013). Sigma-1 Receptor Antagonism as Opioid Adjuvant Strategy: Enhancement of Opioid Antinociception without Increasing Adverse Effects. Eur. J. Pharmacol..

[136] Bruna J., Videla S., Argyriou A.A., Velasco R., Villoria J., Santos C., Nadal C., Cavaletti G., Alberti P., Briani C. (2018). Efficacy of a Novel Sigma-1 Receptor Antagonist for Oxaliplatin-Induced Neuropathy: A Randomized, Double-Blind, Placebo-Controlled Phase IIa Clinical Trial. Neurotherapeutics.

[137] (2016). Discovery Studio 16.

[138] Laggner C., Schieferer C., Fiechtner B., Poles G., Hoffmann R.D., Glossmann H., Langer T., Moebius F.F. (2005). Discovery of High-Affinity Ligands of σ1 Receptor, ERG2, and Emopamil Binding Protein by Pharmacophore Modeling and Virtual Screening. J. Med. Chem..

[139] García M., Virgili M., Alonso M., Alegret C., Fernández B., Port A., Pascual R., Monroy X., Vidal-Torres A., Serafini M.T. (2020). 4-Aryl-1-Oxa-4,9-Diazaspiro[5.5]Undecane Derivatives as Dual μ-Opioid Receptor Agonists and σ1 Receptor Antagonists for the Treatment of Pain. J. Med. Chem..

[140] García M., Virgili M., Alonso M., Alegret C., Farran J., Fernández B., Bordas M., Pascual R., Burgueño J., Vidal-Torres A. (2020). Discovery of EST73502, a Dual μ-Opioid Receptor Agonist and σ1 Receptor Antagonist Clinical Candidate for the Treatment of Pain. J. Med. Chem..

[141] Fulgenzi G., Graciotti L., Faronato M., Soldovieri M.V., Miceli F., Amoroso S., Annunziato L., Procopio A., Taglialatela M. (2006). Human Neoplastic Mesothelial Cells Express Voltage-Gated Sodium Channels Involved in Cell Motility. Int. J. Biochem. Cell Biol..

[142] Kim F.J., Maher C.M. (2017). Sigma1 Pharmacology in the Context of Cancer. Handb. Exp. Pharmacol..

[143] Brackenbury W.J., Djamgoz M.B.A., Isom L.L. (2008). An Emerging Role for Voltage-Gated Na+ Channels in Cellular Migration: Regulation of Central Nervous System Development and Potentiation of Invasive Cancers. Neuroscientist.

[144] Abate C., Niso M., Abatematteo F.S., Contino M., Colabufo N.A., Berardi F. (2020). PB28, the Sigma-1 and Sigma-2 Receptors Modulator With Potent Anti–SARS-CoV-2 Activity: A Review About Its Pharmacological Properties and Structure Affinity Relationships. Front. Pharmacol..

[145] Riganas S., Papanastasiou I., Foscolos G.B., Tsotinis A., Dimas K., Kourafalos V.N., Eleutheriades A., Moutsos V.I., Khan H., Margarita P. (2012). New Adamantane Derivatives with Sigma Affinity and Antiproliferative Activity. Med. Chem..

[146] Gordon D.E., Jang G.M., Bouhaddou M., Xu J., Obernier K., White K.M., O’Meara M.J., Rezelj V.V., Guo J.Z., Swaney D.L. (2020). A SARS-CoV-2 Protein Interaction Map Reveals Targets for Drug Repurposing. Nature.

[147] Berardi F., Ferorelli S., Abate C., Colabufo N.A., Contino M., Perrone R., Tortorella V. (2004). 4-(Tetralin-1-Yl)- and 4-(Naphthalen-1-Yl)Alkyl Derivatives of 1-Cyclohexylpiperazine as σ Receptor Ligands with Agonist σ 2 Activity. J. Med. Chem..

[148] Pati M.L., Hornick J.R., Niso M., Berardi F., Spitzer D., Abate C., Hawkins W. (2017). Sigma-2 Receptor Agonist Derivatives of 1-Cyclohexyl-4-[3-(5-Methoxy-1,2,3,4-Tetrahydronaphthalen-1-Yl)Propyl]Piperazine (PB28) Induce Cell Death via Mitochondrial Superoxide Production and Caspase Activation in Pancreatic Cancer. BMC Cancer.

[149] Liu C.C., Yu C.F., Wang S.C., Li H.Y., Lin C.M., Wang H.H., Abate C., Chiang C.S. (2019). Sigma-2 Receptor/TMEM97 Agonist PB221 as an Alternative Drug for Brain Tumor. BMC Cancer.

[150] Niso M., Abate C., Contino M., Ferorelli S., Azzariti A., Perrone R., Colabufo N.A., Berardi F. (2013). Sigma-2 Receptor Agonists as Possible Antitumor Agents in Resistant Tumors: Hints for Collateral Sensitivity. ChemMedChem.

[151] Pati M.L., Niso M., Ferorelli S., Abate C., Berardi F. (2015). Novel Metal Chelators Thiosemicarbazones with Activity at the σ2 Receptors and P-Glycoprotein: An Innovative Strategy for Resistant Tumor Treatment. RSC Adv..

[152] Pati M.L., Niso M., Spitzer D., Berardi F., Contino M., Riganti C., Hawkins W.G., Abate C. (2018). Multifunctional Thiosemicarbazones and Deconstructed Analogues as a Strategy to Study the Involvement of Metal Chelation, Sigma-2 (σ2) Receptor and P-Gp Protein in the Cytotoxic Action: In Vitro and in Vivo Activity in Pancreatic Tumors. Eur. J. Med. Chem..

[153] Abate C., Niso M., Lacivita E., Mosier P.D., Toscano A., Perrone R. (2011). Analogues of σ Receptor Ligand 1-Cyclohexyl-4-[3-(5-Methoxy-1,2,3,4- Tetrahydronaphthalen-1-Yl)Propyl]Piperazine (PB28) with Added Polar Functionality and Reduced Lipophilicity for Potential Use as Positron Emission Tomography Radiotracers. J. Med. Chem..

[154] Berardi F., Abate C., Ferorelli S., Uricchio V., Colabufo N.A., Niso M., Perrone R. (2009). Exploring the Importance of Piperazine N-Atoms for σ2 Receptor Affinity and Activity in a Series of Analogs of 1-Cyclohexyl-4-[3-(5-Methoxy-1,2,3,4-Tetrahydronaphthalen-1-Yl)-Propyl]Piperazine (PB28). J. Med. Chem..

[155] Brent P.J., Pang G., Little G., Dosen P.J., Van Helden D.F. (1996). The Sigma Receptor Ligand, Reduced Haloperidol, Induces Apoptosis and Increases Intracellular-Free Calcium Levels [Ca2+]i in Colon and Mammary Adenocarcinoma Cells. Biochem. Biophys. Res. Commun..

[156] Nieto F.R., Cendán C.M., Sánchez-Fernández C., Cobos E.J., Entrena J.M., Tejada M.A., Zamanillo D., Vela J.M., Baeyens J.M. (2012). Role of Sigma-1 Receptors in Paclitaxel-Induced Neuropathic Pain in Mice. J. Pain.

[157] Nieto F.R., Cendán C.M., Cañizares F.J., Cubero M.A., Vela J.M., Fernández-Segura E., Baeyens J.M. (2014). Genetic Inactivation and Pharmacological Blockade of Sigma-1 Receptors Prevent Paclitaxel-Induced Sensory-Nerve Mitochondrial Abnormalities and Neuropathic Pain in Mice. Mol. Pain.

[158] Hashimoto K. (2013). Potential Role of the Sigma-1 Receptor Chaperone in the Beneficial Effects of Donepezil in Dementia with Lewy Bodies. Clin. Psychopharmacol. Neurosci..

[159] Mangiatordi G.F., Intranuovo F., Delre P., Abatematteo F.S., Abate C., Niso M., Creanza T.M., Ancona N., Stefanachi A., Contino M. (2020). Cannabinoid Receptor Subtype 2 (CB2R) in a Multitarget Approach: Perspective of an Innovative Strategy in Cancer and Neurodegeneration. J. Med. Chem..

[160] Abate C., Ferorelli S., Contino M., Marottoli R., Colabufo N.A., Perrone R., Berardi F. (2011). Arylamides Hybrids of Two High-Affinity σ 2 Receptor Ligands as Tools for the Development of PET Radiotracers. Eur. J. Med. Chem..

